# Exergaming Interventions for Preventing Falls and Injurious Falls in Older People: Systematic Review and Meta-Analysis of Randomized Controlled Trials

**DOI:** 10.2196/89807

**Published:** 2026-07-31

**Authors:** Charlotte Eost-Telling, Lisa McGarrigle, Chunhu Shi, Annemarie Money, Yang Yang, Kimberly Lazo Green, Saima Ahmed, Rachel Christie, Abodunrin Aminu, Kim Delbaere, Eling D de Bruin, Emma Stanmore, Chris Todd

**Affiliations:** 1 Division of Nursing Midwifery and Social Work, Faculty of Biology, Medicine and Health, National Institute for Health and Care Research (NIHR) Applied Research Collaboration Greater Manchester, University of Manchester, Manchester, England, United Kingdom, 44 161 306 1247; 2 Manchester Academic Health Science Centre, Manchester, England, United Kingdom; 3 Division of Nursing Midwifery and Social Work, Faculty of Biology, Medicine and Health, National Institute for Health and Care Research, Policy Research Unit in Older People and Frailty / Healthy Ageing, University of Manchester, Manchester, England, United Kingdom; 4 School of Nursing and Healthcare, Atlantic Technological University, Castlebar, County Mayo, Ireland; 5 Falls, Balance and Injury Research Centre, Neuroscience Research Australia, Randwick, New South Wales, Australia; 6 School of Health Sciences, University of New South Wales, Kensington, New South Wales, Australia; 7 Department of Health, University of Applied Sciences St. Gallen, St. Gallen, Saint Gallen, Switzerland; 8 Department of Health Sciences and Technology, Motor Control and Learning Group - Institute of Human Movement Sciences and Spor, ETH Zurich, Zurich, Zurich, Switzerland; 9 Department of Neurobiology, Care Sciences, and Society, Division of Physiotherapy, Karolinska Institutet, Huddinge, Stockholm, Sweden; 10 Manchester University NHS Foundation Trust, Manchester, England, United Kingdom

**Keywords:** exergames, active video gaming, video games, virtual reality, exercise, physical activity, aging, fall prevention, accidental falls

## Abstract

**Background:**

Exergames, which combine physical exercise with interactive gameplay, are increasingly being incorporated into fall prevention programs for older adults. Gamified elements, such as real-time feedback and progress tracking, may enhance motivation, engagement, and adherence. Although several systematic reviews have examined the effects of exergaming on balance and physical function, fewer have focused specifically on clinically meaningful outcomes, such as falls and injurious falls, or on indicators that may influence real-world adoption of exergames.

**Objective:**

This study aimed to evaluate the effectiveness of exergaming interventions for preventing falls and injurious falls in people aged ≥60 years and to synthesize evidence on implementation-related outcomes, including adherence, acceptability, concerns about falling, quality of life, adverse events, and cost-effectiveness.

**Methods:**

MEDLINE, Embase, CINAHL Plus, PsycINFO, and the Cochrane Central Register of Controlled Trials (CENTRAL) were searched from inception to February 2025 for randomized controlled trials evaluating exergaming interventions in older adult populations across all settings. Outcomes included fall rate, number of fallers and injurious falls, and implementation-related secondary outcomes. Risk of bias was assessed using RoB 2.0, and certainty of evidence was assessed using Grading of Recommendations Assessment, Development, and Evaluation (GRADE). Data were synthesized narratively and, where appropriate, pooled using meta-analysis.

**Results:**

Nine studies (N=1385) met the inclusion criteria. Comparator-specific analyses suggested that exergaming may reduce fall rates compared with active intervention comparators, although the magnitude and certainty of effect varied, and substantial heterogeneity was present across analyses. Moderate-certainty evidence also suggested that exergames reduced the number of older adults experiencing one or more falls at 12-month follow-up compared with usual care (risk ratio 0.75, 95% CI 0.61‐0.92). Evidence for injurious falls, quality of life, concerns about falling, adherence, acceptability, and cost-effectiveness was limited or inconsistent. When pooled across all control groups, exergaming interventions were associated with a lower overall fall rate than comparator interventions (incidence rate ratio 0.53, 95% CI 0.41‐0.68), although substantial heterogeneity was present (*I*²=76%).

**Conclusions:**

Low- to moderate-certainty evidence suggests that exergames may reduce fall rates, particularly in comparisons with active intervention control groups, and may reduce the number of fallers compared with usual care. These findings indicate that exergaming may offer a useful adjunct to established fall prevention strategies for older adults, particularly where sustained engagement with conventional exercise is challenging. However, substantial heterogeneity, modest sample sizes, and limited long-term follow-up reduce confidence in these estimates, and more rigorous, large-scale trials are needed before routine implementation can be recommended. This review extends previous exergaming syntheses by focusing on clinically meaningful outcomes, including falls and injurious falls, while also considering implementation-related factors relevant to real-world uptake.

## Introduction

### Rationale

Each year, approximately one-quarter to one-third of adults aged >65 years living in the community experience a fall, a finding consistently reported across international populations [1-3]. A fall is defined as “an unexpected event in which the participant comes to rest on the ground, floor, or lower level” [4]. The consequences of falls range in severity from minor bruising to debilitating fractures or even death [5]. Beyond physical injuries, falls can also have considerable psychosocial effects, as concerns about falling and loss of independence can lead to reduced social engagement and diminished quality of life (QoL) [6].

Falls also place a substantial financial burden on health care systems worldwide due to hospitalizations and the need for long-term care following injurious falls [7]. Many risk factors for falls, including balance and strength deficits, cognitive decline, and reduced muscle mass, are associated with aging and low levels of physical activity [8]. Regular physical activity can mitigate these issues by improving muscle strength, balance, and overall physical function, as well as enhancing cognitive performance, mood, and QoL [9-11]. Strong evidence suggests that physical activity programs, particularly those incorporating balance and functional exercises, can reduce both the rate of falls and the number of older people who experience falls in the community [12]. Nevertheless, adherence to these programs is often modest and tends to decline over time, with systematic reviews reporting full adherence rates as low as 21% for home-based programs [13] and only approximately half of participants remaining adherent at 12 months [14,15].

Exergaming offers a promising alternative by providing an immersive and engaging experience, incorporating gamified elements such as feedback, achievements, and progress tracking to enhance motivation and adherence [16]. Exergames, broadly defined as active video games that integrate gameplay with physical exercise, use real-time motion detection to track players’ movements [17]. These games may be delivered via animated gaming platforms or within 2D or 3D virtual reality (VR) environments and are increasingly being explored as a tool for fall prevention [18,19].

As exergaming can be tailored to an individual’s functional ability, it may make physical activity more accessible, enjoyable, and acceptable for older adults, addressing barriers such as low motivation and physical limitations [16,20]. As a fall prevention tool, exergaming interventions often gamify physical activities targeting balance, strength, 3D movement (eg, dance), endurance [20-22], and cognitive function [23,24]. These interventions can be provided to individuals or groups in a wide range of settings, including home-based settings, community settings, and hospitals, with or without facilitation by a health professional.

Several systematic reviews and meta-analyses have examined exergaming in older adults, reporting improvements in intermediate outcomes, such as balance, physical function, cognitive outcomes, and concerns about falling [25-32]. These findings suggest that exergaming may improve important fall-related risk factors and may enhance exercise engagement through interactive feedback and gamification. However, comparatively less attention has been given to whether these interventions translate into reductions in clinically measured falls and injurious falls, which remain the most meaningful outcomes for patients, clinicians, and health services. In addition, previous reviews have given limited consideration to implementation-relevant factors, such as adherence, acceptability, adverse events, and cost-effectiveness, all of which are important when evaluating the real-world feasibility of integrating exergaming into fall prevention pathways. Furthermore, prior syntheses have rarely distinguished between usual care, evidence-based active exercise controls, and non–evidence-based active comparator interventions, limiting the interpretation of whether exergaming offers benefit beyond established fall prevention practice. A more clinically focused synthesis of the available evidence is therefore needed.

### Objectives

To address these gaps, the aim of this systematic review and meta-analysis was to evaluate the effectiveness of exergaming interventions for preventing falls and injurious falls in adults aged ≥60 years. The secondary objectives were to synthesize evidence relating to QoL, concerns about falling, balance confidence, adherence, acceptability, adverse events, and cost-effectiveness and to examine findings according to comparator type.

## Methods

### Overview

We followed Cochrane systematic review methods [33]. The review was conducted and reported in accordance with the PRISMA (Preferred Reporting Items for Systematic Reviews and Meta-Analyses) 2020 statement, using the PRISMA 2020 expanded checklist [34], the PRISMA 2020 for Abstracts checklist [35], and the PRISMA-S extension for reporting literature searches [36]. The study protocol was prospectively registered with PROSPERO (CRD42020214721).

### Information Sources and Search Strategy

Searches were completed first in March 2023 and updated using the same search strategy in February 2025. The following databases were searched from inception to this date: MEDLINE (Ovid), Embase (Ovid), CINAHL Plus (EBSCO), PsycINFO (Ovid), and Cochrane Central Register of Controlled Trials (CENTRAL) (Ovid). The PICOS framework was used to develop eligibility criteria around the themes of exergames, falls, and older people (Table 1). The search strategy combined database-specific controlled vocabulary terms (eg, MeSH, Emtree, CINAHL Headings, and APA Thesaurus) with free-text terms. Full search strategies for all databases, including database-specific adaptations, are provided in Supplementary Material S1 in Multimedia Appendix 1. No date restrictions were applied, but searches were limited to studies published in English in accordance with the eligibility criteria. Where published reports lacked sufficient methodological or outcome detail, study authors were contacted for clarification or to request additional data. In addition to database searches, reference lists of included studies and relevant systematic reviews were screened, and forward citation searching was conducted manually by 2 independent reviewers (CE-T and YY) using Google Scholar to identify any additional eligible studies.

**Table 1.** Inclusion and exclusion criteria.

Eligibility criterion	Inclusion	Exclusion
Population	Sample was predominantly aged ≥60 y (operationalized as mean age minus one SD >60 where necessary) All health conditions	Sample was predominantly aged <60 y
Interventions	All types of exergaming interventions All delivery methods	Nonexergaming intervention
Control	No intervention or usual care Active intervention not believed to reduce falls Active intervention considered likely to reduce falls	None
Outcomes	Primary: falls or injurious falls Secondary: health-related quality of life, concern about falling or fear of falling, balance confidence, adherence and/or acceptability, adverse events, cost-effectiveness	Studies where the only outcomes relate to balance, functional mobility, and/or strength
Setting	All settings	None
Types of studies	Randomized controlled trials Cluster randomized controlled trials	Nonrandomized studies Observational, quasi-experimental, qualitative
Aim	Main purpose is to investigate the effectiveness of exergaming on falls or injurious falls in older people	Main purpose is not to investigate the effectiveness of exergaming on falls or injurious falls in older people
Type of publication	Peer-reviewed journal publication	Not a peer-reviewed journal publication Conference abstract, theses, letter to editor, reviews, descriptive (editorials, books, and reports), and protocol only
Language	English language	Other languages
Dates	All dates	None

### Eligibility Criteria

We included randomized controlled trials from any setting that evaluated the effects of exergaming interventions on falls or injurious falls in older adults aged ≥60 years (Table 1). This threshold aligns with international definitions of older populations (eg, World Health Organization [2]) and allows inclusion of a broad range of relevant studies. We included trials with generally healthy participants and participants with conditions that may increase the risk of falls (eg, dementia, stroke, Parkinson disease, and frailty).

We included trials in which the main purpose of the study was to investigate the effectiveness of exergaming. We excluded studies that focused solely on the design of exergaming interventions.

### Interventions and Comparators

The review considered all types of exergaming interventions and all delivery methods. This included all interactive active games and immersive or nonimmersive VR platforms, and interventions of any length and duration were eligible. Control interventions included usual care, standard exercise programs with an evidence base in fall prevention (eg, strength and balance training), as well as those with no evidence base in fall prevention (eg, an educational leaflet on exercise and low-intensity or low-frequency exercise) [12].

The types of exercise primarily targeted by the intervention were categorized according to the Prevention of Falls Network Europe (ProFaNE) taxonomy [37], as follows: (1) gait, balance, coordination, and functional training; (2) strength or resistance training; (3) flexibility; (4) 3D exercise (eg, Tai Chi or dance); (5) general physical activity; (6) endurance (eg, treadmill walking); and (7) other kinds of exercise.

### Outcomes

The primary outcomes of interest were (1) rate of falls (number of falls over a period and falls per person-year), (2) number of fallers (number of people experiencing one or more falls), and (3) number of people who experienced one or more injurious falls [38].

The secondary outcomes were (1) health-related QoL (measured using a validated scale such as the EQ-5D [39]); (2) concern about falling (measured using a validated scale such as the Falls Efficacy Scale-International [40]); (3) balance confidence (measured using a validated scale such as the Activities-Specific Balance Confidence Scale [41]); (4) adherence to the intervention (defined as the extent to which participants completed the prescribed exergaming program, including attendance, exercise frequency, duration, or retention); (5) acceptability of the intervention (defined as participants’ perceptions of usability, satisfaction, enjoyment, and willingness to engage with or continue the program, as reported by study authors); (6) adverse events (defined as any negative outcome resulting either directly or indirectly from the assigned treatment [42], measured as the number of people who experienced one or more adverse events); and (7) cost-effectiveness of the intervention. These outcome definitions were based on measures reported in the included studies.

### Protocol Registration and Amendments

This review was prospectively registered in PROSPERO (CRD42020214721) and conducted in accordance with the registered protocol. One methodological amendment was made during the review process. We initially planned to combine all active intervention comparators into a single analysis; however, important conceptual and clinical differences were identified between evidence-based fall prevention interventions and non–evidence-based active controls. These comparator groups were therefore analyzed separately to improve the interpretability and clinical relevance of the findings. This decision was made prior to final data synthesis. No other substantive deviations from the registered protocol were made.

### Study Selection

Titles and abstracts from the database searches were imported into the Rayyan web app for systematic reviews [43], and duplicate records were removed. Records were then screened for relevance by 2 independent reviewers (selected from a pool of CE-T, LM, EDB, ES, KD, YY, AM, KG, RC, and AA), and full texts of studies considered potentially relevant were assessed for eligibility. The reasons for exclusion at the full-text stage were recorded and are reported in Supplementary Material S2 in Multimedia Appendix 1. The same prespecified eligibility criteria (Table 1) were applied at title or abstract and full-text screening, and the table served as the screening framework for reviewers. No automated decision-making tools were used, and all decisions were made by reviewers. Any disagreements were resolved through discussion with a third independent reviewer from the pool.

### Data Extraction

Data were extracted from included studies by 2 independent reviewers (KG and YY) using a standardized data extraction form in an Excel (Microsoft) spreadsheet, and an independent reviewer (CE-T or AA) checked for accuracy. Extracted data included author details, country, year of publication, participant characteristics (eg, age, gender, and health status), study characteristics (eg, design, location, setting, sample size, and length of follow-up), intervention details (eg, exergaming device and dose), outcomes measured, and key results.

We used PROGRESS-Plus criteria (place of residence, race/ethnicity/culture/language, occupation, gender/sex, religion, education, socioeconomic status, and social capital) [44] when extracting participant characteristics.

The TIDieR (Template for Intervention Description and Replication) checklist was used to describe the included interventions and controls [45].

### Risk of Bias Assessment

The Excel version of the Cochrane risk of bias (RoB 2.0) tool was used to assess the risk of bias [46,47]. Two independent reviewers assessed the risk of bias for each study, and any disagreements were resolved through discussion with an additional reviewer. Risk of bias was classified as high, low, or some concerns, across the following domains: randomization process, deviations from intended interventions, missing outcome data, measurement of the outcome, and selection of the reported result.

### Reporting Bias Assessment

Reporting bias, including publication bias and selective reporting, was considered during synthesis. Formal statistical assessment (eg, funnel plots or Egger test) was not undertaken because of the small number of studies included in each meta-analysis, making such methods unreliable. Potential reporting bias was therefore considered qualitatively when interpreting the findings.

### Assessment of the Certainty of Evidence

We conducted GRADE assessments on the overall evidence for each primary outcome, split by comparator group [48]. Two independent reviewers assessed each outcome (CE-T and AM or LM and SA), and any disagreements were resolved through discussion. Overall bias per outcome was based on the majority rating across domains.

### Effect Measures and Data Synthesis

Findings were synthesized narratively and summarized in overview tables to facilitate comparison across interventions. Where 2 or more studies contributed sufficiently clinically comparable data, pooled overall or comparator-specific meta-analyses were conducted in Review Manager (RevMan; version 5.4) [49]. Outcomes informed by single studies are presented for consistency of effect estimation but interpreted narratively. Incidence rate ratios (IRRs) with 95% CIs were calculated for fall rate outcomes, risk ratios (RRs) with 95% CIs for binary outcomes (number of fallers and number of participants experiencing one or more injurious falls), mean differences (MDs) for continuous outcomes reported on the same scale, and standardized MDs (SMDs) where different measurement scales were used. Fixed effects or random effects models were selected according to the clinical comparability between studies. Where pooling was not appropriate because of limited study numbers, heterogeneous outcome measures, or inconsistent reporting, results were synthesized narratively in accordance with Synthesis Without Meta-analysis guidance [50].

### Subgroup Analysis

Planned subgroup analysis for the primary outcomes included participant age (older vs younger populations), health condition (eg, healthy, stroke, Parkinson disease, and frailty), and intervention setting (community, residential care home, hospital, assisted care facility, sheltered housing, and retirement community). However, these analyses were not feasible because too few studies contributed data within each subgroup. Comparator-specific analyses were undertaken instead, as these provided the most clinically interpretable basis for exploring variation in effect estimates.

## Results

### Study Selection

**Figure 1.**
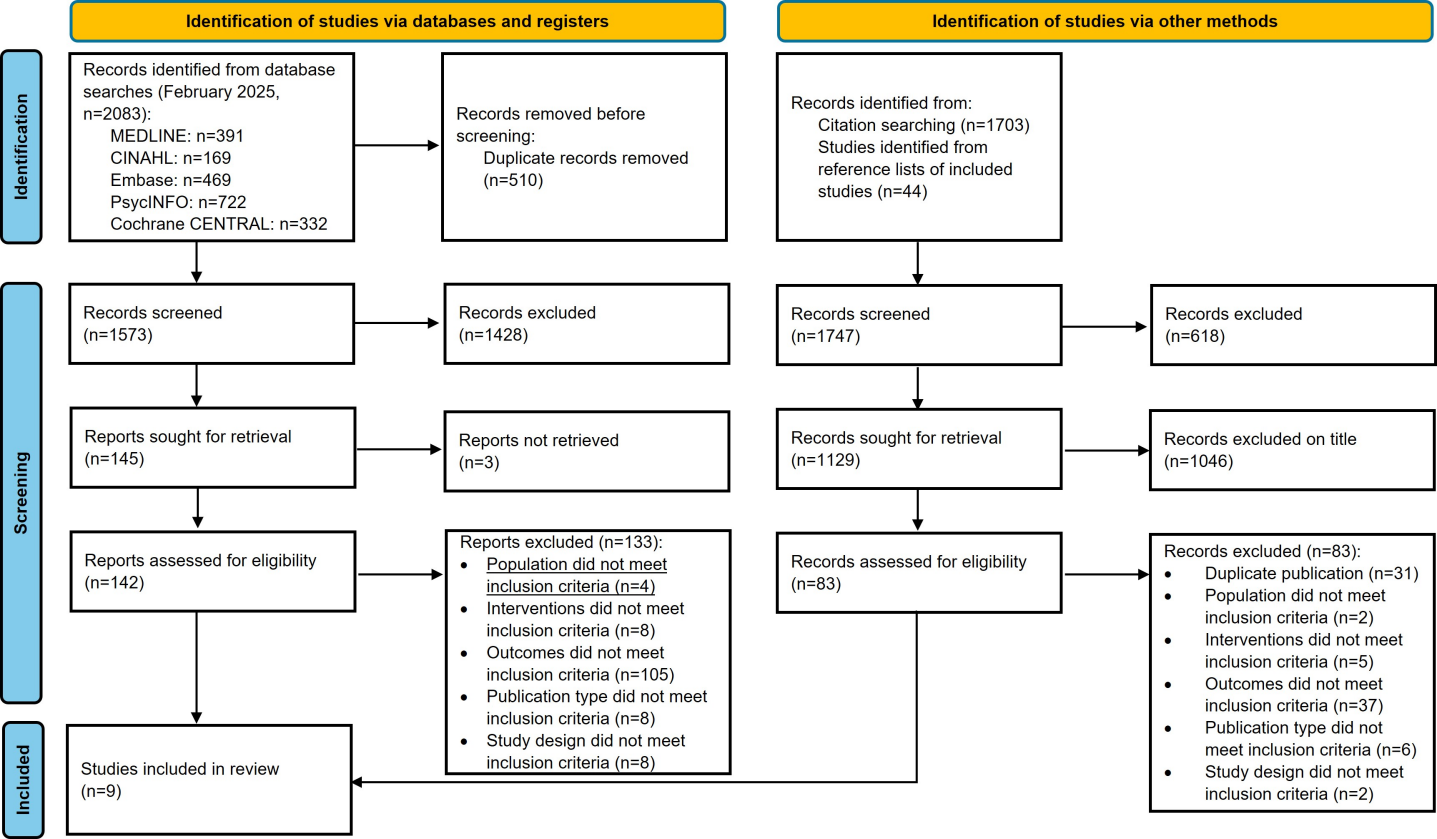
PRISMA (Preferred Reporting Items for Systematic Reviews and Meta-Analyses) flowchart of the systematic review process.

We identified 3830 records, and after removal of duplicates, 3320 (86.7%) were screened on title and abstract, and 225 (5.9%) full-text reports were assessed for eligibility. Of these, 216 (96%) full-text reports were excluded, most commonly because of ineligible study design, inappropriate intervention, ineligible population, or absence of relevant fall outcomes. A full list of studies excluded at the full-text screening, together with reasons for exclusion, is provided in Supplementary Material S2 in Multimedia Appendix 1. Nine randomized controlled trials involving 1385 participants met the inclusion criteria, with 51% (701/1385) in intervention groups and 49% (684/1385) in control groups. Studies varied considerably in participant populations, intervention formats, comparator types, and follow-up duration (Figure 1).

### Participant Characteristics

Participant characteristics and PROGRESS-Plus aspects of equity are presented in Table 2. In total, 59% (817/1385) were women (intervention groups: 416/817, 51% and control groups: 401/817, 49%). The mean age of participants was 72.8 (SD 7.9; intervention groups: 72.9, SD 7.8, and control groups: 72.6, SD 8.0) years. The mean age of participants in the no contact or usual care control studies was 72.8 (SD 6.9) years; in the active control studies (evidence based), the mean age was 73.1 (SD 9.0) years; and in the active control studies (non–evidence based), the mean age was 72.6 (SD 7.9) years. Four studies recruited participants based on a diagnosis of Parkinson using clinical criteria [51-54]. Participants were recruited in 3 studies on the basis that they had a history of falls [51,53,55], and 1 study specifically recruited participants with mild cognitive impairment [53].

**Table 2.** Participant characteristics.

Author, year	Participant characteristics				PROGRESS-Plus^a^ measures
	n	Age (y), mean (SD)	Gender (female), n (%)	Clinical condition	
Control group: usual care or no intervention					
Song et al, 2018 [54] (N=53)	I^b^: 28; C^c^: 25	I: 68 (7); C: 65 (7)	I: 16 (52); C: 20 (69)	Idiopathic Parkinson disease	P^d^
Stanmore et al, 2019 [56] (N=92)	I: 49; C: 43	I: 77.9 (8.9); C: 77.8 (10.2)	I: 45 (80.4); C: 38 (76.0)	None reported	P, R^e^, O^f^, SES^g^, SC^h^
Sturnieks et al, 2024 [57] (N=507)	I: 252; C: 255	I: 72.6 (5.7); C: 72.5 (5.5)	I: 178 (70.6); C: 182 (71.4)	None reported (excluded people with neurological, acute psychiatric, or cognitive impairment)	P, E^i^, SC
Control group: evidence-based active control					
Fu et al, 2015 [55] (N=60)	I: 30; C: 30	I: 82.4 (3.8); C: 82.3 (4.3)	I: 19 (63.3); C: 20 (66.6)	One or more falls in the past year	P
Gandolfi et al, 2017 [52] (N=70)	I: 36; C: 34	I: 67.5 (7.2); C: 69.8 (9.4)	I: 15 (39.5); C: 10 (26.3)	Parkinson disease (modified Hoehn and Yahr stages 2.5‐3)	P
Kwok et al, 2016 [58] (N=80)	I: 40; C: 40	I: 70.5 (6.7); C: 69.8 (7.5)	I: 36 (90); C: 30 (80)	43 participants had a history of falls in the past year; mild-to-moderate physical frailty (9‐5 points) on the short physical performance battery	P, R, L, SC
Control group: non–evidence-based active control					
Alagumoorthi et al, 2022 [51] (N=192)	I: 96; C: 96	I: 69.7 (10); C: 68.5 (9.8)	I: 45 (46.9); C: 33 (34.4)	Idiopathic Parkinson disease (Hoehn-Yahr stage 2.5‐4). History of at least three falls in previous 3 mo	P
Eggenberger et al, 2015 [59] (N=49)	I: 24; C: 25	I (dance): 77.3 (6.3); C: 80.8 (4.7)	I (dance): 14 (58.3); C: 16 (64.0)	None reported	P
Mirelman et al, 2016 [53] (N=282)	I: 146; C: 136	I: 74.2 (6.9); C: 73.3 (6.4)	I: 48 (33); C: 52 (38)	Self-reported 2 or more falls in the past 6 mo; some with MCI^j^ (0.5 on Clinical Dementia Rating scale) or Parkinson disease (Hoehn and Yahr stage II-III)	P, E

a PROGRESS-Plus: place of residence, race/ethnicity/culture/language, occupation, gender/sex, religion, education, socioeconomic status, and social capital.

b I: intervention.

c C: control.

d P: place of residence.

e R: race, ethnicity, or culture.

f O: occupation.

g SES: socioeconomic status.

h SC: social capital.

i E: education.

j MCI: mild cognitive impairment

No study included data on the research locale (eg, urban or rural), 2 studies noted race, ethnicity, or culture [56,58], and 1 study each presented data on the participants’ language [58], occupation [56], number of years in education [53], and socioeconomic status [56]. Three studies included some information on aspects, which could contribute to social capital (ie, living arrangements) [56-58].

### Study and Intervention Characteristics

#### Overview

A summary of study and intervention characteristics is presented in Table 3. Studies were conducted across 11 countries, including 1 study each from India [51], Switzerland [59], China [55], Italy [52], Singapore [58], the United Kingdom [56], Australia or New Zealand [54], and Australia [57], and 1 study from a multicountry study (Belgium, Israel, Italy, the Netherlands, and the United Kingdom) [53]. Study settings included 2 clinics: a clinical center [53] and a geriatric clinic [59]. The remaining studies were conducted across hospital and community settings [51], in both community and university laboratory settings [54], in a satellite center, and at home [58], and 4 in community settings, such as the home [52], an assisted living facility [56], a nursing home [55], and 1 unspecified community location [57].

**Table 3.** Study and intervention characteristics of included randomized controlled trials.

Author, year	Setting	Intervention	Duration or frequency	Devices	Type of exercise	Type of control	Length of follow-up	Outcomes assessed
Control group: usual care control								
Song et al, 2018 [54]	C^a^	Home-based exergame step training: stepping on central panel to match onscreen directional cues. Accuracy feedback and scores were provided after each round.	15 min per session, 3 times a week for 12 wk	“Dance Dance Revolution” Stepmania	iv^b^	UC^c^: continue with usual health care	3 mo	Falls, CF^d^, ADV^e^
Stanmore et al, 2019 [56]	LTCF^f^	Tailored strength and balance exergames: standardized strength and balance exergames individually prescribed and progressed based on ability (eg, increased difficulty, duration, or number of games).	30 min per session, 3 times a week for 12 wk	Microsoft Kinect and tailored software	i^g^	UC: leaflets on fall prevention advice	3 mo	Falls, HQoL^h^, CF, ADH^i^, ACC^j^, ADV, C/CE^k^
Sturnieks et al, 2024 [57]	C	Smart±step program: participants stepped on a Bluetooth-connected mat to match directional targets. Games trained speed, accuracy, motor control, and cognitive skills (working memory, visuospatial skills, dual-tasking, inhibition, and attention).	120‐150 min per wk for 12 mo	Smart±step computer game system and step mat	i	UC: health information brochure	12 mo	Falls, HQoL, CF, BC^l^, ADH, ACC, ADV
Control group: evidence-based active control								
Fu et al, 2015 [55]	LTCF	Wii Fit balance training: balance training games (Soccer Heading, Table Tilt, and Balance Bubble)	1 h per session, 3 times a week for 6 wk	Nintendo Wii Fit balance board	i	AC^m^: conventional balance training, including lower limb muscle strengthening exercises	12 mo	Falls
Gandolfi et al, 2017 [52]	C	Tele-Wii (home-based virtual reality telerehabilitation): 10 Wii exergames remotely supervised and progressed by a physiotherapist based on each patient’s clinical status and improvement	50 min per session, 3 times per week for 7 consecutive weeks (21 sessions)	Nintendo Wii Fit	i and vii^n^	AC: sensory integration balance training of balance and gait exercises	1 mo	Falls, HQoL, BC, ACC, C/CE
Kwok et al, 2016 [58]	C	Nintendo Wii exercise program: WiiActive Balance Board and resistance band training, incorporating cardiovascular, strengthening, calisthenics, and balance training. Sessions included 20 min each of Wii training, individualized exercises, and home exercise	1 h per session, 2‐3 times a week for 12 wk	Nintendo Wii Active	i, ii^o^, iii^p^, iv, and vii	AC: traditional gym exercise	3 and 6 mo	Falls, CF, ADH, ACC, ADV
Control group: non–evidence-based active control								
Alagumoorthi et al, 2022 [51]	C	Wii Sports-based strategy training: 8 games, selected based on validated movement analyses to target key balance strategies and movements needed to prevent falls	30‐40 min per session, 3 times per week for 12 wk	Nintendo Wii console	i	AC: traditional balance training	3 mo	Falls, HQoL
Eggenberger et al, 2015 [59]	C	VR^q^ dance-based aerobic training (DANCE): 1×1 m platform with 4 pressure-sensitive zones that detected steps forward, backward, left, and right. Followed arrow cues on a screen, with feedback on accuracy	1 h per session, twice per week for 6 mo	Impact Dance Platforms, Stepmania	i and iv	AC: treadmill walking (PHYS) and treadmill walking with verbal memory exercise (MEMORY)	12 mo	Falls, CF
Mirelman et al, 2016 [53]	C	Treadmill training with virtual reality: real-time foot-tracking projected onto a virtual environment while walking on a treadmill, with added cognitive challenges (eg, attention, planning, dual-tasking)	45 min per session, 3 times a week for 6 wk	Modified Microsoft Kinect with nonimmersive VR and treadmill	vi	AC: treadmill training	6 mo	Falls, HQoL, ADH, ADV

a C: community.

b iv: 3D exercise (eg, Tai Chi or dance).

c UC: usual care.

d CF: concerns about falling.

e ADV: adverse event.

f LTCF: long-term care facility.

g i: gait, balance, coordination, and functional task training.

h HQoL: health-related quality of life.

i ADH: adherence.

j ACC: acceptability.

k C/CE: costs/cost-effectiveness.

l BC: balance confidence.

m AC: active control.

n vii: other kinds of exercise, which improve the correct use of ankle and hip strategy during static conditions.

o ii: strength or resistance training (alternating biceps curl).

p iii: flexibility.

q VR: virtual reality.

The interventions in the included studies were described using the TIDieR checklist (see Supplementary Material S3 in Multimedia Appendix 1 for details). A range of exergaming and VR systems were used across studies: 4 used the Nintendo Wii platform, 1 used Microsoft Kinect with tailored software, 1 used a modified Microsoft Kinect combined with nonimmersive VR and treadmill training, 2 used dance-based systems (Konami Dance Dance Revolution or Positive Gaming Impact Dance Platform) paired with a Stepmania mat, and 1 used bespoke stepping software with a pressure-sensitive mat [57].

In terms of the control group, 3 studies included usual care or no intervention [54,56,57], 3 included an evidence-based exercise fall prevention program [52,55,58], and 3 used a non–evidence-based program [51,53,59]. Active controls included balance training (with or without physiotherapy) [51,52,55], treadmill training [53,59], or gym-based exercises [58].

#### Intervention Dosage and Delivery Characteristics

The active exergame dose ranged from 6 to 52 weeks, with a frequency of 2 to 3 sessions per week, where specified. Session duration ranged from 15 minutes to 1 hour. One study gave an overall number of minutes per week to be completed but did not specify how many sessions should be undertaken or how long each should last [57].

All studies involved support for the exergame sessions provided by physiotherapists, health care professionals, or trained postgraduate students. Seven interventions included supervised sessions throughout the study [51-53,55,56,58,59], while the remaining 2 provided supervision during the initial setup and training session, with ongoing technical support and advice available as needed for the remainder of the study [54,57]. Studies did not report on the type and frequency of help needed by participants to undertake the exergame exercises.

#### Risk of Bias and Certainty of the Evidence

**Figure 2.**
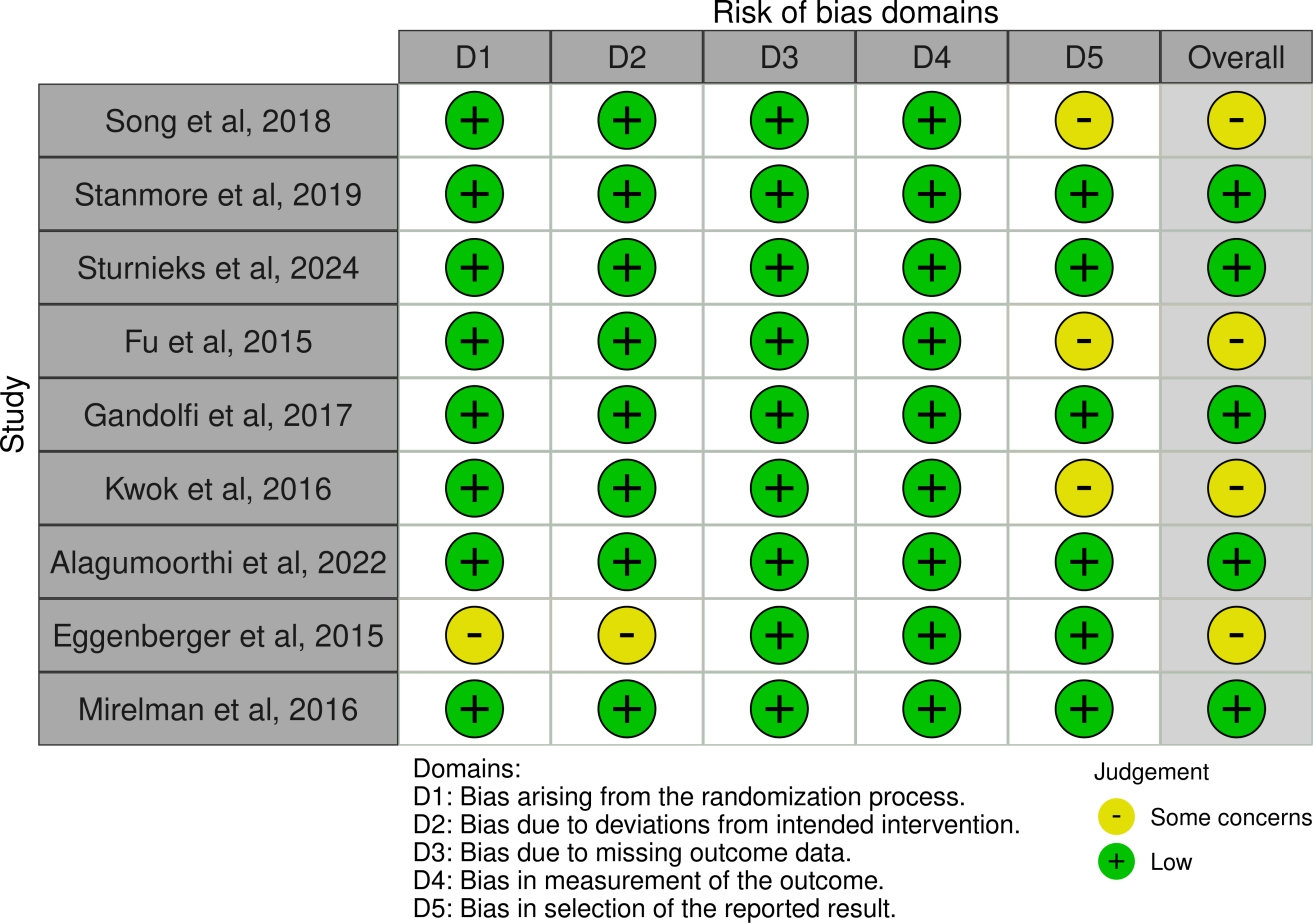
Risk of bias summary assessments [51-59].

Risk-of-bias (RoB 2) summary assessments are shown in Figure 2.

Due to the nature of the intervention, no studies were able to blind participants to their group assignment. Nevertheless, 8 studies were single blinded with assessors blinded to the participant group [51-55,57-59], and the final study was nonblinded because the cluster design revealed participants’ allocation due to their assisted living facility [56]. Four studies had some additional concerns over the risk of bias: 3 over the selection of the reported result because there was a lack of information about whether the data that were used to produce the results had been analyzed following a prespecified analysis plan, which had been finalized before the unblinded outcome data were available for analysis [54,55,58]. The fourth study had some concerns over a lack of information on the randomization process and deviations from the intended intervention [59].

Certainty of evidence (GRADE) for each primary outcome is presented in Table 4, with full assessment details provided in Supplementary Material S4 in Multimedia Appendix 1. Key drivers for downgrading of outcome certainty were imprecision and inconsistency across studies.

**Table 4.** Overview of Grading of Recommendations Assessment, Development, and Evaluation assessments by primary outcome.

Primary outcome	Comparison 1: usual care or no control	Comparison 2: evidence-based active control	Comparison 3: non–evidence-based active control
Rate of falls	⨁⨁◯◯ (low)	⨁⨁◯◯ (low)	⨁⨁⨁◯ (moderate)
Number of fallers	⨁⨁◯◯ (low)	⨁⨁◯◯ (low)	⨁⨁◯◯ (low)
Number of people experiencing injurious falls	⨁⨁◯◯ (low)	N/A^a^	N/A

a N/A: not applicable.

### Effectiveness of Interventions

The effectiveness of exergaming interventions is presented according to comparator type: usual care, evidence-based active controls, and non–evidence-based active controls. This approach was used to distinguish the effects of exergaming against usual care or no intervention, against established exercise programs with evidence for fall prevention, and against active control activities not specifically designed to reduce falls.

### Comparison 1: Exergames Versus Usual Care or No Intervention Control Group

**Figure 3.**
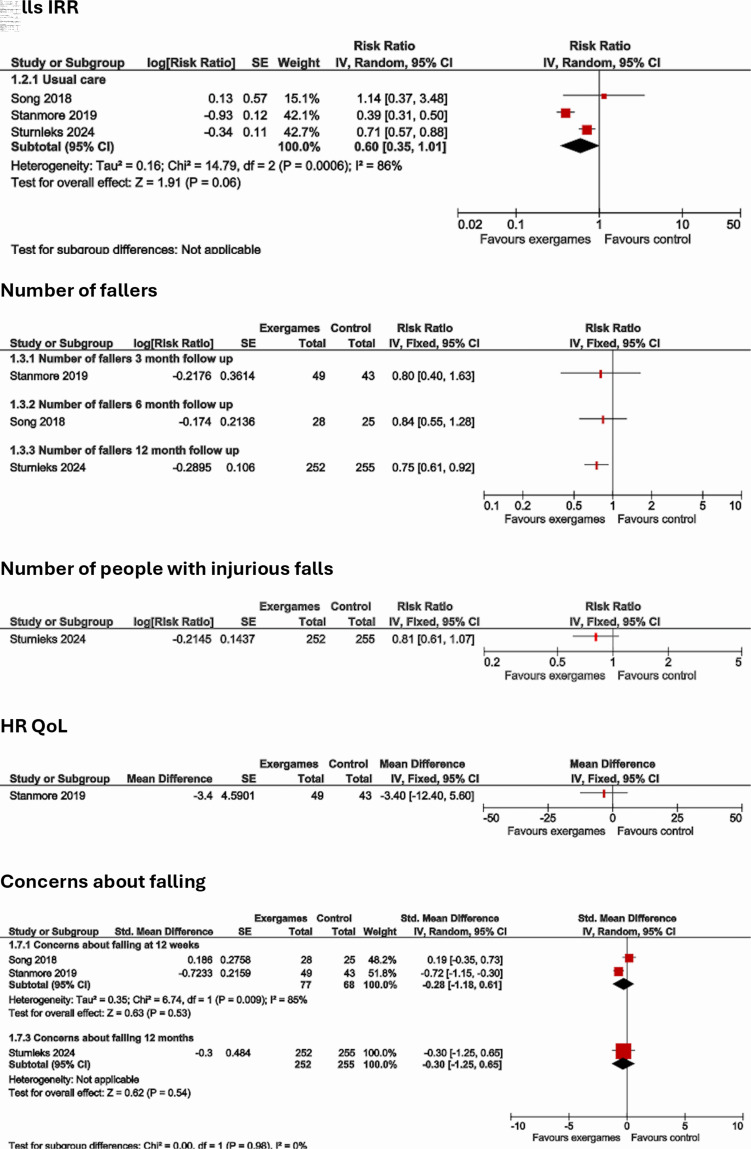
Forest plots of exergames versus usual care [54,56,57]. HR: health related; IRR: incidence rate ratio; QoL: quality-adjusted life year.

Three studies, including 652 participants, compared the use of exergames against usual care or no intervention [54,56,57]. Forest plots for this comparison are presented in Figure 3.

#### Primary Outcomes

##### Rate of Falls: IRR

All 3 studies reported fall rates, which were converted to IRR for comparison (n=652) [54,56,57]. Using a random-effects model, the pooled estimate suggested a possible reduction in fall rate (low certainty evidence). However, the CI crossed the line of no effect, indicating uncertainty in the effect estimate. Substantial heterogeneity was present, and this finding should therefore be interpreted with caution (IRR 0.60, 95% CI 0.35‐1.01; *I*^2^=86%). Heterogeneity was evident in participants, settings, and intervention components, and one small study, which included participants with Parkinson disease, showed no significant result with wide CIs [54].

##### Number of Fallers (Number of People Experiencing One or More Falls)

Three studies (n=652), 2 at low risk of bias and 1 with some concerns, presented data on the number of fallers (low certainty evidence): one each at 3-month follow-up [56], 6-month follow-up [54], and a 12-month follow-up [57]. No significant differences were found at 3-month or 6-month follow-up (RR 0.80, 95% CI 0.40‐1.63 and RR 0.84, 95% CI 0.55‐1.28, respectively). At 12-month follow-up, exergames may result in a reduction in the number of fallers (RR 0.75, 95% CI 0.61‐0.92).

##### Number of People Who Experienced One or More Injurious Falls

One study (n=507) [57] showed exergaming may reduce the relative risk of people experiencing one or more injurious falls by an average of 19% (low certainty evidence). This estimate was imprecise, ranging from a 39% reduction to an increase of 8% (RR 0.81, 95% CI 0.61‐1.08).

### Secondary Outcomes

#### Quality of Life

One study (n=92) reported on QoL using the EQ-5D-5L VAS scale [56]. The evidence suggests that exergames do not improve QoL at 3-month follow-up (MD −3.40, 95% CI −12.40 to 5.60).

#### Effects on Concerns About Falling

Three studies (n=652) reported on the effect of exergaming interventions on concerns about falling [54,56,57]. Two studies reported this outcome at 12-week follow-up [54,56] and the third study at 6- and 12-month follow-up [57]. For this study, we focused on the 12-month data, as it represented the longest follow-up period. The evidence from a meta-analysis of the 12-week follow-up data suggests that exergames result in no difference in concerns about falling (SMD −0.28, 95% CI −1.18 to 0.61), and there was high heterogeneity between the studies (*I*^2^=85%). Evidence from the study with the 12-month follow-up period [57] also suggested that exergames did not reduce concerns about falling (SMD −0.10, 95% CI −0.23 to 0.02).

#### Balance Confidence

No study reported on balance confidence in this comparison.

#### Acceptability

Two studies (n=599) included acceptability measures [56,57]. The System Usability Scale [60] mean score in the first study was 82.4 (SD 15.5) at 12-week follow-up [56]. The second study measured the System Usability Scale at 6 and 12 months and found a mean score of 80.4 (SD 15.8) at 6-month and 83.3 (SD 13.9) at 12-month follow-up [57]. At all measurement points in both studies, scores were above 80, which is considered excellent and indicates good usability. The Technology Acceptance Model [61] was included in one paper [56] at 12-week follow-up, and overall, all domains were considered high or very high, but with some variation in behavioral intention and perceived usefulness: easy to use, 6.3 (SD 1.4); useful, 5.9 (SD 1.9); favorable attitude, 6.6 (SD 1.2); and intention to use, 5.7 (SD 2.2).

#### Adherence

All 3 studies reported on retention rates, with a median of 87% and a range from 81% to 91.7% [54,56,57]. The median adherence rate to the minimum prescribed dose of exercise was 69%, ranging from 50.8% to 85%. However, 1 study also noted that only 0.6% of participants achieved the target of 120 minutes of exercise per week over the 12-month intervention period [57].

#### Adverse Events

No studies reported serious adverse events as a result of the intervention: 2 reported no adverse events from the intervention [56,57] and the third study reported 1 noninjurious fall while completing the exergame step training under the guidance of an experienced physiotherapist [54].

#### Cost-Effectiveness

One study (n=92) included the cost-effectiveness of the exergame intervention [56]. The authors reported a point estimate incremental cost-effectiveness ratio of £15,209.80 (US $20,239.71) per quality-adjusted life year (QALY). Using 1000 bootstrap replications, they found there was a 61% probability that exergames were cost-effective at the lower National Institute for Health and Care Excellence threshold of £20,000 (US $26,624.08) per QALY, rising to 73% at the upper threshold of £30,000 (US $39,970) per QALY.

### Comparison 2: Exergames Versus Evidence-Based Active Control

**Figure 4.**
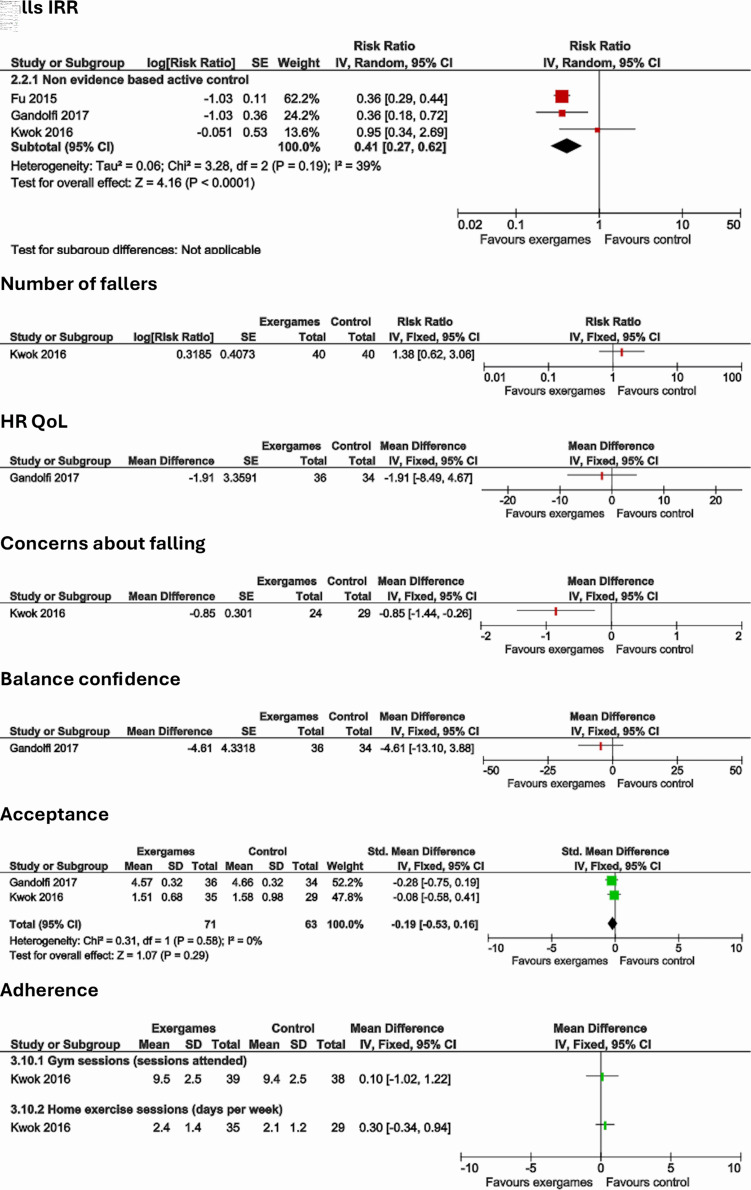
Forest plots of exergames versus evidence-based active controls [52,55,58]. HR: health related; IRR: incidence rate ratio; QoL: quality-adjusted life year.

Three studies (n=210) reported on the use of exergame interventions and an evidence-based active control [52,55,58]. Forest plots for this comparison are presented in Figure 4.

#### Primary Outcomes

##### Rate of Falls: IRR

The IRRs were calculated for all studies, and the evidence from a meta-analysis suggests that exergames result in a reduction in fall rate (IRR 0.41, 95% CI 0.27‐0.62; *I*^2=^39%; low certainty evidence).

##### Number of Fallers (Number of People Experiencing One or More Falls)

A single study (n=80) reported intervention effects on the number of fallers at 12-month follow-up [58]. The evidence, which was of low certainty, showed that exergames result in little to no difference in the number of fallers (RR 1.38, 95% CI 0.62‐3.06).

##### Number of People Who Experienced One or More Injurious Falls

No studies reported on this outcome.

### Secondary Outcomes

#### Quality of Life

One study (n=70) examined the effects of an exergame intervention on QoL using the EQ5D scale [52]. Findings indicated that exergames do not increase QoL compared to evidence-based active controls (MD −1.91, 95% CI −8.49 to 4.67).

#### Concerns About Falling

Concerns about falling were reported in one study (n=80), using the Modified Falls Efficacy Scale [58]. The evidence suggests exergames result in a reduction in concerns about falling at 12-week follow-up (MD 0.80, 95% CI 0.61‐0.99).

#### Balance Confidence

Balance confidence was reported in 1 study (n=70), using the Activities-Specific Balance Confidence scale [52]. Exergames may result in little to no difference in balance confidence at 1-month follow-up (MD −4.61, 95% CI −13.10 to 3.88).

#### Acceptability

Two studies included measures of acceptability (n=150) [52,58]. Evidence from a meta-analysis of these studies suggests that exergames result in little or no difference in acceptability (SMD −0.19, 95% CI −0.53 to 0.16 *I*^2^=0%).

#### Adherence

One study (n=80) reported on adherence rates [58], assessed through attendance at intervention sessions and the frequency of home exercises performed. Attendance data suggest little to no difference in adherence (MD 0.10, 95% CI −1.02 to 1.22). The home exercise compliance (number of days per week completed over the 1-y follow-up) also suggested no difference in adherence (MD 0.30, 95% CI −0.34 to 0.90).

#### Adverse Events

Two studies included adverse events [52,58], but both reported no adverse events during the studies or follow-up periods.

#### Cost Data

Cost-effectiveness was not included in any study; however, one study did consider cost (n=70) [52]. The cost of rehabilitation was calculated as €383.55 (US $439.20) per intervention group participant (€23,299.00 [US $26,679.22] for the whole intervention group) and €602.10 (US $689.45) per control group participant (€28,899.80 [US $33,092.58] for the whole control group).

### Comparison 3: Exergames Versus Active Intervention (Non–Evidence Based)

**Figure 5.**
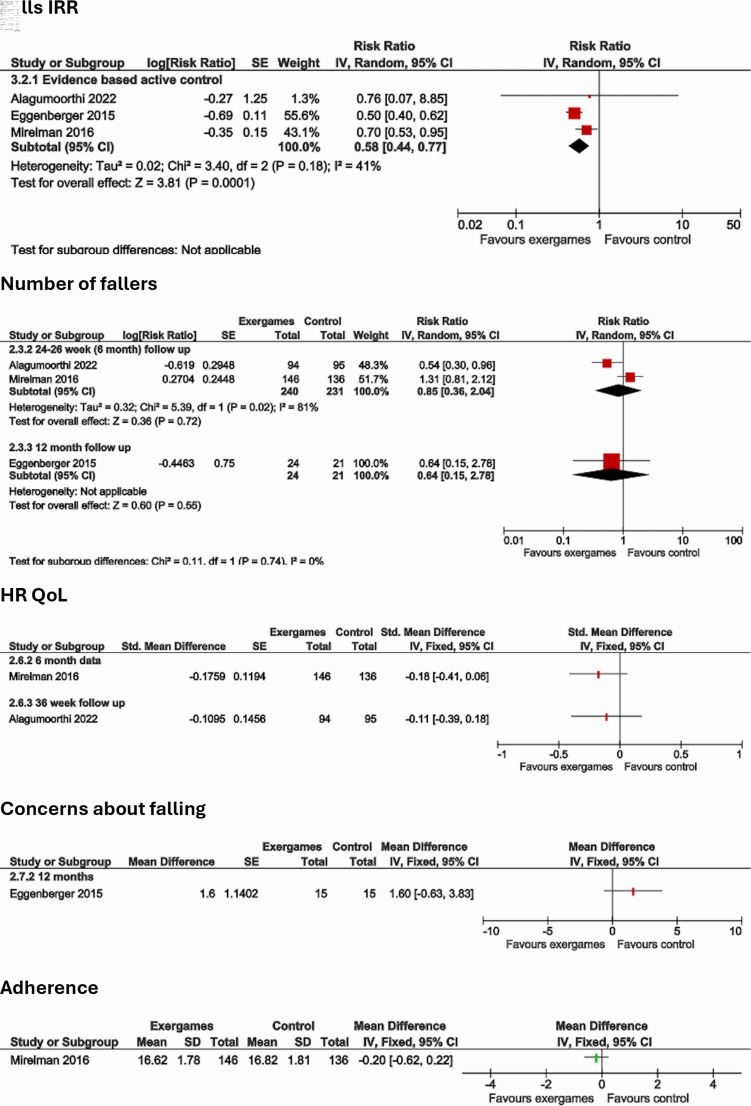
Forest plots of exergames versus non–evidence-based active controls [51,53,59]. HR: health related; IRR: incidence rate ratio; QoL: quality-adjusted life year.

Three studies (n=523) reported on the use of exergame interventions and a non–evidence-based active control [51,53,59]. Forest plots for this comparison are presented in Figure 5.

#### Primary Outcomes

##### Rate of Falls: IRR

All 3 studies reported the effects of exergame interventions on the fall rate in this comparison [51,53,59]. Two reported follow-ups at 6 months (n=474) [51,53] and one at 12 months (n=49) [59]. These data were converted to IRR for comparison, and the moderate-certainty evidence from a meta-analysis indicates that exergames likely result in a reduction in fall rate (IRR 0.58, 95% CI 0.44‐0.77; *I*^2^=41%).

##### Number of Fallers (Number of People Experiencing One or More Falls)

Three studies (n=523) reported the number of people experiencing one or more falls [51,53,59]. At 6-month follow-up, evidence from a meta-analysis of 2 studies (n=474) suggests no difference in the number of fallers (RR 0.85, 95% CI 0.36‐2.04) [51,53]. At 12-month follow-up, low-certainty evidence indicated that exergames did not reduce the number of fallers (RR 0.64, 95% CI 0.15‐2.68) [59].

##### Number of People Who Experienced 1 or More Injurious Falls

No studies reported on this outcome

### Secondary Outcomes

#### Quality of Life

QoL was measured in 2 studies (n=474) using either the 36-Item Short Form Health Survey (SF-36) [62] at 6-month follow-up [53] or the Parkinson’s Disease Questionnaire (PDQ-39) summary index [63] at 36-week follow-up [51]. Evidence from the studies suggests that exergames result in little or no difference in QoL.

#### Concerns About Falling

One study (n=49) reported on concerns about falling using the FES-I scale [59]. The evidence is very uncertain about the effect of exergames on concerns about falling (MD 1.60, 95% CI −0.63 to 3.83).

#### Balance Confidence

No studies in this comparison group reported on this outcome.

#### Acceptability

No studies in this comparison reported on this outcome.

#### Adherence

Evidence from a single study (n=282) [53] was very uncertain about the effect of exergames on adherence (MD −0.20, 95% CI −0.62 to 0.22).

#### Adverse Events

Two studies reported on adverse events [51,53]. Although overall 24 events were reported, none occurred because of the study.

#### Cost-Effectiveness

No studies in this comparison reported on this outcome measure.

#### Overall Falls Pooled Effect Across Comparator Groups

An exploratory pooled analysis across all comparator groups, with low- to medium-certainty evidence, suggested that exergaming interventions were associated with a lower fall rate than comparator interventions (IRR 0.53, 95% CI 0.41‐0.68). However, substantial heterogeneity was present (*I*²=76%), indicating considerable variation in effect estimates across studies, and therefore, this pooled effect should be interpreted cautiously. Comparator-specific analyses are likely to provide more clinically meaningful insights, as the magnitude and certainty of effect differed across control categories. Differences in participant populations, intervention characteristics, comparator content, and follow-up duration are likely to have contributed to the observed heterogeneity.

## Discussion

### Summary of Findings

This review synthesized evidence on the effectiveness of exergaming interventions for fall prevention in older adults by examining falls and injurious falls as primary outcomes alongside implementation-relevant secondary outcomes. Comparator-specific analyses suggest that exergaming interventions may reduce fall rates when compared with usual care and some active comparators, although the magnitude and certainty of effect varied across comparisons. When data were pooled across all comparator groups, exergaming was also associated with an overall reduction in fall rate; however, this broad summary estimate should be interpreted cautiously because substantial heterogeneity indicates important variation between intervention contexts, participant groups, and comparator conditions. Moderate-certainty evidence also indicated that exergames may reduce the number of older adults experiencing one or more falls at 12-month follow-up compared with usual care. However, evidence for injurious falls was limited to a single study, and confidence in several pooled estimates was reduced by substantial heterogeneity, small numbers of contributing studies, and imprecision.

These findings suggest that exergaming may have value as an adjunct to established exercise-based fall prevention approaches, particularly where motivation, adherence, and sustained participation in conventional exercise programs are challenging. At the same time, the review highlights that exergaming interventions are not homogeneous, and effectiveness is probably influenced by intervention design, level of supervision, participant health status, comparator type, and follow-up duration.

Evidence for secondary outcomes, including QoL, concerns about falling, balance confidence, adherence, acceptability, and cost-effectiveness, was limited, of low certainty, or inconsistently reported. Adverse events were uncommon and were not clearly attributed to the interventions, but the small number of studies reporting implementation outcomes limits broader conclusions regarding long-term feasibility, sustainability, and economic value. Overall, these findings suggest that exergaming may offer a promising additional approach within fall prevention; however, the current evidence base is not yet robust enough to support widespread routine adoption.

### Comparison With Prior Work

Previous systematic reviews have generally reported beneficial but mixed effects of exergaming on intermediate outcomes, such as balance, mobility, physical function, concerns about falling, and cognitive performance, but have offered limited synthesis of clinically meaningful fall outcomes [64-74]. This review extends this literature by focusing specifically on falls and injurious falls, by examining implementation-related outcomes relevant to real-world delivery, and by considering effects according to comparator type. This provides a more clinically relevant understanding of where exergaming may offer a benefit within fall prevention pathways.

Our findings are broadly consistent with previous reviews in suggesting that exergames can be effective, but they also confirm that these effects are not uniform and may depend on intervention design, comparator type, and duration of follow-up. Previous reviews, including Chen et al [75], have reported short-term improvements were more commonly seen than long-term effects, and this was evident in our included studies. This highlights the importance of longer follow-up periods, in line with ProFaNE recommendations, to establish whether short-term gains translate into sustained reductions in falls over time.

Variation in game design, exercise challenge, alignment with the Systems Framework for Postural Control [76], progression, feedback, and professional supervision may also explain differences in effectiveness across studies. Standardizing key intervention components and clearer reporting of intervention dose and support needs would improve comparability across future studies and improve confidence in findings [45]. This is particularly relevant given that adherence was stronger in studies with structured supervision, consistent with previous work emphasizing declining motivation in unsupported programs [77].

Economic and implementation evidence is also limited. Only 2 included studies reported intervention costs and only one considered cost-effectiveness [56], reflecting the limited economic evaluation seen across the wider exergaming literature [78,79]. Similarly, acceptability and usability were also underreported, despite their importance for long-term use of digital interventions among older adults [16,77,80,81]. Together, these gaps indicate that future exergaming studies should move beyond efficacy measures alone and address whether exergames represent a viable and scalable fall prevention approach for real-world adoption [82,83].

### Strengths and Limitations

This review has several strengths, including a comprehensive multi-database search, prospective protocol registration, comparator-stratified analyses, and use of established tools including RoB 2.0 and GRADE. However, several limitations should be considered.

Our search was limited to studies published in English, which may have introduced language bias. There was also considerable clinical and methodological heterogeneity across studies in participant populations, intervention design, comparators, outcome reporting, and follow-up duration, which limited the precision and generalizability of pooled estimates. Exergame interventions varied widely in the types of equipment used, the extent to which games were purpose designed and the degree to which they were based on known effective strength and balance exercises (eg, Otago [84]), the amount of supervision by physiotherapists or health care professionals, and the duration and frequency of delivery. Furthermore, in several studies, exergames were delivered as part of a wider package of support, making it difficult to isolate the individual contribution of the exergame.

Notably, the studies involving support did not report on the amount or types of support needed by participants. Future research should therefore collect and report this information to better understand its impact on outcomes and to help assess the cost of delivery of the intervention in the real world.

Participant health status also varied considerably: 4 studies included people living with Parkinson disease or mild cognitive impairment, and 3 studies specifically recruited people with a history of falls. Such comorbidities, known to affect strength, balance, and falls, may have impacted the study outcomes [85-88]. Although we planned to conduct subgroup analyses, including those based on health status and PROGRESS-Plus, as outlined in the PROSPERO protocol, the relatively small number of studies included in meta-analyses limited the feasibility of more extensive subgroup analyses and formal assessment of publication bias. Many of the studies also had small sample sizes, limiting their ability to detect intervention effects and increasing the risk of type II error. While pooled “overall” estimates are presented, these should be interpreted with caution, and subgroup analyses by comparator type are likely to provide more clinically meaningful insights.

In addition, pooled estimates were calculated using the standard DerSimonian-Laird random effects approach in RevMan, which, although widely used, may provide less conservative interval estimates than alternative methods such as Hartung-Knapp-Sidik-Jonkman when only a small number of studies are available. Follow-up periods ranged from 1 to 12 months but were often relatively short, with 22% of studies including a follow-up of less than 6 months, and 33% at 6 months. Given that the ProFaNE recommends a minimum follow-up of 12 months in fall prevention trials, because these interventions may produce delayed effects, requiring sustained adherence over time to demonstrate efficacy [12], the longer-term sustainability of exergaming effects remains uncertain.

### Implications for Policy and Practice

The findings of this review suggest that exergames may play a role as a complementary component of fall prevention programs for older adults, particularly given the evidence that they may reduce fall rates and the number of people experiencing a fall when compared with usual care. However, the current evidence does not support exergames as a stand-alone replacement for established exercise-based fall prevention interventions.

While the included studies varied considerably in intervention design and dosage, several interventions associated with favorable outcomes incorporated structured, progressive exercise delivered multiple times per week over several weeks or months. This is broadly consistent with established fall prevention exercise principles, which emphasize sufficient frequency, intensity, and progression [12,89]. Given the heterogeneity of interventions and limited evidence on optimal dosage, specific recommendations for exergaming interventions cannot yet be made, and further research is needed to determine the most effective training parameters. However, given the variability in intervention design and delivery, standardized guidelines for exergame design and delivery could be developed to ensure greater consistency across interventions. Purpose-designed serious exergames with appropriate theoretical underpinnings should be developed to unlock the full potential of exergame-enhanced fall prevention interventions [90]. This would facilitate the integration of exergaming with established exercise-based fall prevention programs such as Otago [91] or FaME [92], potentially optimizing effectiveness.

Adherence remains a recognized challenge. Most studies reported good adherence rates when structured support, such as supervision by physiotherapists, was provided, suggesting policies promoting supported or remotely monitored delivery may improve engagement and effectiveness [77]. Ensuring equitable access will therefore require investment in user-friendly technology, digital literacy programs, and technical support to reduce barriers to sustained participation among older adults [93-95].

Finally, our review highlights the need for longer follow-up periods and more robust economic evaluations before exergames can be confidently integrated into routine service delivery. Given the preliminary evidence suggesting cost-effectiveness within National Institute for Health and Care Excellence thresholds, further evaluations are needed to confirm these findings. Policymakers should consider further evaluations to guide future resource allocation and determine whether exergames represent a financially sustainable addition to health care and social care fall prevention pathways.

### Conclusions

This review suggests that exergaming interventions may help reduce falls among older adults, particularly in comparisons against active intervention control groups, while pooled analyses also indicate potential overall benefit across diverse intervention settings. However, these findings should be interpreted cautiously because effect estimates varied considerably between studies and were limited by substantial heterogeneity, modest sample sizes, and relatively few long-term follow-up assessments.

Evidence for injurious falls and implementation-relevant outcomes, such as adherence, acceptability, QoL, and cost-effectiveness, remains limited or inconsistently reported. As such, although exergaming appears to offer a potentially engaging and clinically relevant adjunct to existing fall prevention programs, the evidence base is not yet sufficiently robust to support widespread routine implementation. Larger, methodologically rigorous trials with standardized fall reporting and longer follow-up are needed to determine which exergaming formats, doses, and delivery models are most effective for sustained fall prevention in older adults.

## Supplementary material

10.2196/89807 Multimedia Appendix 1 Supplementary material, including full search strategy, excluded paper list, TIDieR checklist, and GRADE assessment.

10.2196/89807 Checklist 1 PRISMA checklist.

## Abbreviations

GRADE: Grading of Recommendations Assessment, Development, and Evaluation
IRR: incidence rate ratio
MD: mean difference
PRISMA: Preferred Reporting Items for Systematic Reviews and Meta-Analyses
PROGRESS-Plus: place of residence, race/ethnicity/culture/language, occupation, gender/sex, religion, education, socioeconomic status, and social capital
QALY: quality-adjusted life year
QoL: quality of life
RR: risk ratio
SMD: standardized mean difference
TIDieR: Template for Intervention Description and Replication
VR: virtual reality
