# Supplementary material for: Exergaming Interventions for Preventing Falls and Injurious Falls in Older People: Systematic Review and Meta-Analysis of Randomized Controlled Trials
Source: JMIR Aging. 2026 Jul 31;9:e89807. doi: 10.2196/89807 (PMC13427072; doi:10.2196/89807)
Supplement: Multimedia Appendix 1 [file aging-v9-e89807-s001.pdf]

Supplementary File 1 – Full search strategy and list of relevant reviews

A Database and trial registry search (searches completed 28.02.25)

Search terms used to identify studies for the systematic review "Exergaming interventions for preventing falls and injurious falls in older people: a systematic review and meta-analysis of randomised controlled trials" for the following electronic databases:

a) Ovid MEDLINE(R) and Epub Ahead of Print, In-Process & Other Non-Indexed Citations, Daily and Versions(R)
b) Embase (OvidSP)
c) CINAHL (EBSCO Plus)
d) PsycINFO (OvidSP)
e) Cochrane Central Register of Controlled Trials (CENTRAL)

a) Ovid MEDLINE(R) and Epub Ahead of Print, In-Process & Other Non-Indexed Citations, Daily and Versions(R)

1. exergam*.mp
2. Telerehabilitation/
3. Telemedicine/
4. Video Game/
5. Virtual Reality/
6. Nintendo wii.mp
7. Xbox Kinect.mp
8. Wii fit.mp
9. balance board.mp
10. eHealth.mp
11. mHealth.mp
12. computers/
13. or/1- 12
14. exercise/
15. physical activity.mp
16. training.mp
17. or/14-16
18. fall*.mp
19. Accidental Falls/
20. injurious fall*.mp

21. fall injury.mp
22. Quality of Life/
23. fear of fall*.mp
24. adherence.mp
25. Compliance/
26. Treatment Adherence and Compliance/
27. drop-out.mp
28. retention.mp
29. uptake.mp
30. Costs and Cost Analysis/
31. or/18-30
32. older people.mp
33. older adult*.mp
34. Aging/
35. elderly.mp
36. Aged/
37. senior*.mp
38. gerontological.mp
39. geriatrics/
40. or/32-39
41. randomi?ed controlled trial.mp
42. 13 and 17 and 31 and 40 and 41

b) Embase (OvidSP)

1. exergam*.mp
2. Telerehabilitation/
3. Telemedicine/
4. Video Game/
5. Virtual Reality/
6. Nintendo wii.mp
7. Xbox Kinect.mp
8. Wii fit.mp
9. balance board.mp
10. eHealth.mp
11. mHealth.mp
12. computers/
13. or/1- 12
14. exercise/
15. physical activity.mp
16. training.mp
17. or/14-16
18. fall*.mp
19. Accidental Falls/
20. injurious fall*.mp
21. fall injury.mp
22. Quality of Life/
23. fear of fall*.mp
24. adherence.mp
25. Compliance/
26. Treatment Adherence and Compliance/
27. drop-out.mp
28. retention.mp
29. uptake.mp
30. Costs and Cost Analysis/

31. or/18-30

32. older people.mp

33. older adult*.mp

34. Aging/

35. elderly.mp

36. Aged/

37. senior*.mp

38. gerontological.mp

39. geriatrics/

40. or/32-39

41. randomi?ed controlled trial.mp

42. 13 and 17 and 31 and 40 and 41

c) CINAHL Plus (EBSCO Plus)

S1	(MH "Exergames")
S2	(MH "Telemedicine")
S3	(MH "Telerehabilitation")
S4	(MH "Video Games")
S5	(MH "Virtual Reality")
S6	(MH "Telehealth")
S7	"mhealth"
S8	"ehealth"
S9	"computer*"
S10	S1 OR S2 OR S3 OR S4 OR S5 OR S6 OR S7 OR S8 OR S9
S11	(MH "Exercise")
S12	(MH "Physical Activity")
S13	"training"
S14	S11 OR S12 OR S13
S15	"fall*"
S16	(MH "Accidental Falls")
S17	"injurious fall*"
S18	"fall injury"
S19	(MH "Quality of Life")
S20	"fear of fall*"
S21	"adherence"
S22	“compliance”
S23	“drop-out”
S24	“retention”
S25	“uptake”
S26	(MH "Costs and Cost Analysis")
S27	S15 OR S16 OR S17 OR S18 OR S19 OR S20 OR S21 OR S22 OR S23 OR S24 OR S25 OR S26
S28	"older people"
S29	"older adult*"

S30	(MH "Aging")
S31	"elderly"
S32	(MH "Aged")
S33	"senior*"
S34	"gerontolog*"
S35	(MH "Geriatrics")
S36	S28 OR S29 OR S30 OR S31 OR S32 OR S33 OR S34 OR S35
S37	(MH "Randomized Controlled Trials")
S38	S10 AND S14 AND S27 AND S36 AND S37

d) PsycINFO (OvidSP)

1. exergam*.mp
2. Telerehabilitation/
3. Telemedicine/
4. Video Game/
5. Virtual Reality/
6. Nintendo wii.mp
7. Xbox Kinect.mp
8. Wii fit.mp
9. balance board.mp
10. eHealth.mp
11. mHealth.mp
12. computers/
13. or/1- 12
14. exercise/
15. physical activity.mp
16. training.mp
17. or/14-16
18. fall*.mp
19. Accidental Falls/
20. injurious fall*.mp
21. fall injury.mp
22. Quality of Life/
23. fear of fall*.mp
24. adherence.mp
25. Compliance/
26. Treatment Adherence and Compliance/
27. drop-out.mp
28. retention.mp
29. uptake.mp
30. Costs and Cost Analysis/

31. or/18-30

32. older people.mp

33. older adult*.mp

34. Aging/

35. elderly.mp

36. Aged/

37. senior*.mp

38. gerontological.mp

39. geriatrics/

40. or/32-39

41. randomi?ed controlled trial.mp

42. 13 and 17 and 31 and 40 and 41

e) Cochrane Central Register of Controlled Trials (CENTRAL)

1. MeSH descriptor: [Exergaming] this term only

2. MeSH descriptor: [Telerehabilitation] this term only

3. MeSH descriptor: [Telemedicine] this term only

4. MeSH descriptor: [Video Games] explode all trees

5. MeSH descriptor: [Virtual Reality] this term only

6. (exergam*).ti,ab,kw

7. (Nintendo wii).ti,ab,kw

8. (Xbox Kinect).ti,ab,kw

9. (Wii fit).ti,ab,kw

10. (balance board).ti,ab,kw

11. (eHealth).ti,ab,kw

12. (mHealth).ti,ab,kw

13. MeSH descriptor: [Computers] this term only

14. MeSH descriptor: [Exercise] this term only

15. (physical activity)ti,ab,kw

16. (training).ti,ab,kw

17. (fall*).ti,ab,kw

18. MeSH descriptor: [Accidental Falls] this term only

19. (injurious fall*). ti,ab,kw

20. (fall injury). ti,ab,kw

21. MeSH descriptor: [Quality of Life] explode all trees

22. (fear of fall*).ti,ab,kw

23. (adherence).ti,ab,kw

24. MeSH descriptor: [Compliance] this term only

25. MeSH descriptor: [Treatment Adherence and Compliance] explode all trees

26. (drop-out).ti,ab,kw

27. (retention).ti,ab,kw

28. (uptake). ti,ab,kw

29. MeSH descriptor: [Costs and Cost Analysis] explode all trees

30. (older people).ti,ab,kw

31. (older adult*).ti,ab,kw

32. MeSH descriptor: [Ageing] this term only

33. (elderly).ti,ab,kw

34. MeSH descriptor: [Aged] explode all trees

35. (senior*).ti,ab,kw

36. (gerontological).ti,ab,kw

37. MeSH descriptor: [Geriatrics] explode all trees

Supplementary File 2 – List of excluded articles with reasons for exclusion

Reference list of studies excluded at full-text with reason for exclusion
Note: A single article may have been excluded for more than one reason, the main reason is given in the table.

Ambrens, M., van Schooten, K.S., Lung, T., Clemson, L., Close, J.C., Howard, K., Lord, S.R., Zijlstra, G.R., Tiedemann, A., Valenzuela, T. and Vandelanotte, C., 2022. Economic evaluation of the e-Health StandingTall balance exercise programme for fall prevention in people aged 70 years and over. Age and ageing, 51(6), p.afac130.	Wrong intervention
Babadi, S.Y. and Daneshmandi, H., 2021. Effects of virtual reality versus conventional balance training on balance of the elderly. Experimental gerontology, 153, p.111498.	Wrong outcomes
Bacha, J.M.R., Gomes, G.C.V., de Freitas, T.B., Viveiro, L.A.P., da Silva, K.G., Bueno, G.C., Varise, E.M., Torriani-Pasin, C., Alonso, A.C., Luna, N.M.S. and D'Andrea Greve, J.M., 2018. Effects of kinect adventures games versus conventional physical therapy on postural control in elderly people: a randomized controlled trial. Games for health journal, 7(1), pp.24-36.	Wrong outcomes
Bakker, J., Donath, L. and Rein, R., 2020. Balance training monitoring and individual response during unstable vs. stable balance Exergaming in elderly adults: Findings from a randomized controlled trial. Experimental gerontology, 139, p.111037.	Wrong outcomes
Rebêlo, F.L., de Souza Silva, L.F., Doná, F., Barreto, A.S. and Quintans, J.D.S.S., 2021. Immersive virtual reality is effective in the rehabilitation of older adults with balance disorders: A randomized clinical trial. Experimental Gerontology, 149, p.111308.	Wrong outcomes
Bergmann, J., Krewer, C., Bauer, P., Koenig, A., Riener, R. and Müller, F., 2017. Virtual reality to augment robot-assisted gait training in non-ambulatory patients with a subacute stroke: a pilot randomized controlled trial. European journal of physical and rehabilitation medicine, 54(3), pp.397-407.	Wrong population
Bieryla, K.A., 2016. Xbox Kinect training to improve clinical measures of balance in older adults: a pilot study. Aging clinical and experimental research, 28(3), pp.451-457.	Wrong outcomes
Blackwood, J., Shubert, T., Fogarty, K. and Chase, C., 2016. The impact of a home-based computerized cognitive training intervention on fall risk measure performance in community dwelling older adults, a pilot study. The journal of nutrition, health & aging, 20(2), pp.138-145.	Wrong outcomes
Blair, C.K., Harding, E., Wiggins, C., Kang, H., Schwartz, M., Tarnower, A., Du, R. and Kinney, A.Y., 2021. A home-based mobile health intervention to replace sedentary time with light physical activity in older cancer survivors: randomized controlled pilot trial. JMIR cancer, 7(2), p.e18819.	Wrong outcomes
Brickwood, K.J., Ahuja, K.D., Watson, G., O'Brien, J.A. and Williams, A.D.,	Wrong outcomes

2021. Effects of activity tracker use with health professional support or telephone counseling on maintenance of physical activity and health outcomes in older adults: randomized controlled trial. JMIR mHealth and uHealth, 9(1), p.e18686.	
Burrows, B.T., Morgan, A.M., King, A.C., Hernandez, R. and Wilund, K.R., 2023. Virtual reality mindfulness and personalized exercise for patients on hemodialysis with depressive symptoms: a feasibility study. Kidney and Dialysis, 3(3), pp.297-310.	Wrong outcomes
Caetano, M.J.D., Menant, J.C., Canning, C.G., Song, J., Schoene, D., Brodie, M. and Lord, S.R., 2016. Effects of videogame step training on gait adaptability in people with Parkinson's disease–A randomized controlled trial: 1857. Movement Disorders, 31, p.S611.	Wrong outcomes
Cano-Mañas, M.J., Collado-Vázquez, S., Rodríguez Hernández, J., Muñoz Villena, A.J. and Cano-De-La-Cuerda, R., 2020. Effects of Video-Game Based Therapy on Balance, Postural Control, Functionality, and Quality of Life of Patients with Subacute Stroke: A Randomized Controlled Trial. Journal of healthcare engineering, 2020(1), p.5480315.	Wrong outcomes
Carrasco-Poyatos, M., Granero-Gallegos, A., López-García, G.D. and López-Osca, R., 2022. HRV-guided training for elders after stroke: a protocol for a cluster-randomized controlled trial. International journal of environmental research and public health, 19(17), p.10868.	Wrong publication type
Chan, W.L.S., Chan, C.W.L., Lam, F.M.H., Chan, H.H.W., Chan, K.C.K., Chan, J.S.K., Chan, O.L.W. and Cheung, D.S.K., 2024. Feasibility, safety, and effects of a Nintendo Ring Fit Adventure™ balance and strengthening exercise program in community-dwelling older adults with a history of falls: A feasibility randomized controlled trial. Geriatrics & Gerontology International, 24, pp.334-341.	Wrong outcomes
Chen, P.Y., Wei, S.H., Hsieh, W.L., Cheen, J.R., Chen, L.K. and Kao, C.L., 2012. Lower limb power rehabilitation (LLPR) using interactive video game for improvement of balance function in older people. Archives of gerontology and geriatrics, 55(3), pp.677-682.	Wrong study design
Cho, K.H., Lee, K.J. and Song, C.H., 2012. Virtual-reality balance training with a video-game system improves dynamic balance in chronic stroke patients. The Tohoku journal of experimental medicine, 228(1), pp.69-74.	Wrong outcomes
Corrêa, F.I., Kunitake, A.I., Segheto, W., Duarte de Oliveira, M., Fregni, F. and Ferrari Corrêa, J.C., 2024. The effect of transcranial direct current stimulation associated with video game training on the postural balance of older women in the community: A blind, randomized, clinical trial. Physiotherapy Research International, 29(1), p.e2046.	Wrong outcomes
de Rooij, I.J., van de Port, I.G., Punt, M., Abbink-van Moorsel, P.J., Kortsmit, M., van Eijk, R.P., Visser-Meily, J.M. and Meijer, J.W.G., 2021. Effect of virtual reality gait training on participation in survivors of subacute stroke: a randomized controlled trial. Physical therapy, 101(5), p.pzab051.	Wrong outcomes

Delbaere, K., Valenzuela, T., Lord, S.R., Clemson, L., Zijlstra, G.R., Close, J.C., Lung, T., Woodbury, A., Chow, J., McInerney, G. and Miles, L., 2021. E-health StandingTall balance exercise for fall prevention in older people: results of a two year randomised controlled trial. bmj, 373.	Wrong intervention
Dockx, K., Alcock, L., Bekkers, E., Ginis, P., Reelick, M., Pelosin, E., Lagravinese, G., Hausdorff, J.M., Mirelman, A., Rochester, L. and Nieuwboer, A., 2017. Fall-prone older people's attitudes towards the use of virtual reality technology for fall prevention. Gerontology, 63(6), pp.590-598.	Wrong outcomes
dos Santos Mendes, F.A., Pompeu, J.E., Lobo, A.M., da Silva, K.G., de Paula Oliveira, T., Zomignani, A.P. and Piemonte, M.E.P., 2012. Motor learning, retention and transfer after virtual-reality-based training in Parkinson's disease–effect of motor and cognitive demands of games: a longitudinal, controlled clinical study. Physiotherapy, 98(3), pp.217-223.	Wrong outcomes
Edwards, J.D., Wadley, V.G., Vance, D.E., Wood, K., Roenker, D.L. and Ball, K.K., 2005. The impact of speed of processing training on cognitive and everyday performance. Aging & mental health, 9(3), pp.262-271.	Wrong outcomes
Ersoy, C. and Iyigun, G., 2021. Boxing training in patients with stroke causes improvement of upper extremity, balance, and cognitive functions but should it be applied as virtual or real?. Topics in stroke rehabilitation, 28(2), pp.112-126.	Wrong outcomes
Fakhro, M.A., Hadchiti, R. and Awad, B., 2020. Effects of Nintendo Wii fit game training on balance among Lebanese older adults. Aging clinical and experimental research, 32(11), pp.2271-2278.	Wrong outcomes
Fanning, J., Brooks, A.K., Hsieh, K.L., Kershner, K., Furlipa, J., Nicklas, B.J. and Rejeski, W.J., 2022. The effects of a pain management-focused mobile health behavior intervention on older adults' self-efficacy, satisfaction with functioning, and quality of life: a randomized pilot trial. International Journal of Behavioral Medicine, 29(2), pp.240-246.	Wrong outcomes
Feng, H., Li, C., Liu, J., Wang, L., Ma, J., Li, G., Gan, L., Shang, X. and Wu, Z., 2019. Virtual reality rehabilitation versus conventional physical therapy for improving balance and gait in Parkinson's disease patients: a randomized controlled trial. Medical science monitor: international medical journal of experimental and clinical research, 25, p.4186.	Wrong outcomes
Gaspar, A.G.M. and Lapão, L.V., 2021. eHealth for addressing balance disorders in the elderly: systematic review. Journal of Medical Internet Research, 23(4), p.e22215.	Wrong study design
Gomes, G.C.V., do Socorro Simões, M., Lin, S.M., Bacha, J.M.R., Viveiro, L.A.P., Varise, E.M., Junior, N.C., Lange, B., Jacob Filho, W. and Pompeu, J.E., 2018. Feasibility, safety, acceptability, and functional outcomes of playing Nintendo Wii Fit PlusTM for frail older adults: A randomized feasibility clinical trial. Maturitas, 118, pp.20-28.	Wrong outcomes
Hale, L.A., Waters, D. and Herbison, P., 2012. A randomized controlled trial to	Wrong

investigate the effects of water-based exercise to improve falls risk and physical function in older adults with lower-extremity osteoarthritis. Archives of physical medicine and rehabilitation, 93(1), pp.27-34.	intervention
Hassett, L., van den Berg, M., Lindley, R.I., Crotty, M., McCluskey, A., van der Ploeg, H.P., Smith, S.T., Schurr, K., Howard, K., Hackett, M.L. and Killington, M., 2020. Digitally enabled aged care and neurological rehabilitation to enhance outcomes with Activity and MObility UsiNg Technology (AMOUNT) in Australia: A randomised controlled trial. PLoS medicine, 17(2), p.e1003029.	Wrong study design
Hastings, S.N., Mahanna, E.P., Berkowitz, T.S., Smith, V.A., Choate, A.L., Hughes, J.M., Pavon, J., Robinson, K., Hendrix, C., Van Houtven, C. and Gentry, P., 2021. Video-enhanced care management for medically complex older adults with cognitive impairment. Journal of the American Geriatrics Society, 69(1), pp.77-84.	Wrong outcomes
Ho, S.F., Thomson, A. and Kerr, A., 2018. 52FEEDBACK INTEGRATED REHABILITATION FOR SIT-TO-STAND TRAINING (FIRST): A PILOT RANDOMISED CONTROLLED TRIAL. Age and Ageing, 47(suppl_3), pp.iii20-iii23.	Wrong outcomes
Imam, B., Miller, W.C., Finlayson, H., Eng, J.J. and Jarus, T., 2017. A randomized controlled trial to evaluate the feasibility of the Wii Fit for improving walking in older adults with lower limb amputation. Clinical rehabilitation, 31(1), pp.82-92.	Wrong outcomes
Jäggi, S., Wachter, A., Adcock, M., de Bruin, E.D., Möller, J.C., Marks, D., Schweinfurther, R. and Giannouli, E., 2023. Feasibility and effects of cognitive–motor exergames on fall risk factors in typical and atypical Parkinson’s inpatients: a randomized controlled pilot study. European journal of medical research, 28(1), p.30.	Wrong outcomes
Jorgensen, M.G., Laessoe, U., Hendriksen, C., Nielsen, O.B.F. and Aagaard, P., 2012. A randomized, double-blind, placebo-controlled trial of Nintendo Wii training on balance impaired older adults. European Geriatric Medicine, 3.	Wrong outcomes
Jørgensen, M.G., 2014. Assessment of postural balance in community-dwelling older adults. Dan Med J [Internet], 61(1), p.B4775.	Wrong study design
Junata, M., Cheng, K.C.C., Man, H.S., Lai, C.W.K., Soo, Y.O.Y. and Tong, R.K.Y., 2021. Kinect-based rapid movement training to improve balance recovery for stroke fall prevention: a randomized controlled trial. Journal of NeuroEngineering and Rehabilitation, 18(1), p.150.	Wrong outcomes
Kang, J.M., Kim, N., Lee, S.Y., Woo, S.K., Park, G., Yeon, B.K., Park, J.W., Youn, J.H., Ryu, S.H., Lee, J.Y. and Cho, S.J., 2021. Effect of cognitive training in fully immersive virtual reality on visuospatial function and frontal-occipital functional connectivity in predementia: randomized controlled trial. Journal of medical Internet research, 23(5), p.e24526.	Wrong outcomes
Kannan, L., Vora, J., Bhatt, T. and Hughes, S.L., 2019. Cognitive-motor exergaming for reducing fall risk in people with chronic stroke: a randomized	Wrong outcomes

controlled trial. NeuroRehabilitation, 44(4), pp.493-510.	
Kanyılmaz, T., Topuz, O., Ardıç, F.N., Alkan, H., Öztekin, S.N.S., Topuz, B. and Ardıç, F., 2022. Effectiveness of conventional versus virtual reality-based vestibular rehabilitation exercises in elderly patients with dizziness: a randomized controlled study with 6-month follow-up. Brazilian Journal of Otorhinolaryngology, 88, pp.S41-S49.	Wrong outcomes
Karssemeijer, E.G., Bossers, W.J., Aaronson, J.A., Sanders, L.M., Kessels, R.P. and Rikkert, M.G.O., 2019. Exergaming as a physical exercise strategy reduces frailty in people with dementia: a randomized controlled trial. Journal of the American Medical Directors Association, 20(12), pp.1502-1508.	Wrong outcomes
Kastner, M., Makarski, J., Hayden, L., Hamid, J.S., Holroyd-Leduc, J., Twohig, M., Macfarlane, C., Hynes, M.T., Prasaud, L., Sklar, B. and Honsberger, J., 2021. Effectiveness of an eHealth self-management tool for older adults with multimorbidity (KeepWell): protocol for a hybrid effectiveness–implementation randomised controlled trial. BMJ open, 11(2), p.e048350.	Wrong outcomes
Kazazi, L., Shati, M., Mortazavi, S.S., Nejati, V. and Foroughan, M., 2021. The impact of computer-based cognitive training intervention on the quality of life among elderly people: a randomized clinical trial. Trials, 22(1), p.51.	Wrong outcomes
Khushnood, K., Altaf, S., Sultan, N., Ali Awan, M.M., Mehmood, R. and Qureshi, S., 2021. Role Wii Fit exer-games in improving balance confidence and quality of life in elderly population. JPMA. The Journal of the Pakistan Medical Association, 71(9), pp.2130-2134.	Wrong outcomes
Kool, J., Oesch, P., Fernandez-Luque, L., Brox, E., Evertsen, G., Civit, A., Hilfiker, R. and Bachmann, S., 2017. AB1222-HPR Exergames versus self-regulated exercises with instruction leaflets to improve adherence in geriatric rehabilitation: a randomized controlled trial. Annals of the Rheumatic Diseases, 76, p.1539.	Wrong outcomes
Kriebs, A., 2024. Home exergame prevents falls. Nature aging, 4(2), pp.167-167.	Wrong publication type
Kwok, B.C., Mamun, K., Chandran, M. and Wong, C.H., 2011. Evaluation of the Frails' Fall Efficacy by Comparing Treatments (EFFECT) on reducing fall and fear of fall in moderately frail older adults: study protocol for a randomised control trial. Trials, 12(1), p.155.	Wrong study design
Lee, G.H., 2019. Effects of Virtual Reality Exercise Program using the Sony PlayStation 2 gaming platform on Balance, Emotion and Quality of Life in Patients with Parkinson's Disease: 132. Movement Disorders, 34, p.S56.	Wrong outcomes
Leppert, F., Siebermair, J., Wesemann, U., Martens, E., Sattler, S.M., Scholz, S., Veith, S., Greiner, W., Rassaf, T., Kääb, S. and Wakili, R., 2021. The INFluence of Remote monitoring on Anxiety/depRession, quality of lifE, and Device acceptance in ICD patients: a prospective, randomized, controlled, single-center trial. Clinical Research in Cardiology, 110(6), pp.789-800.	Wrong outcomes

Lewis, J.B., 2023. In older adults with frailty, virtual reality exercise training improves walking speed and balance. Annals of internal medicine, 176(9), p.JC106.	Wrong study design
Li, S., Li, Y., Liang, Q., Yang, W.J., Zi, R., Wu, X., Du, C. and Jiang, Y., 2022. Effects of tele-exercise rehabilitation intervention on women at high risk of osteoporotic fractures: study protocol for a randomised controlled trial. BMJ open, 12(11), p.e064328.	Wrong study design
Liao, Y.Y., Yang, Y.R., Wu, Y.R. and Wang, R.Y., 2015. Virtual reality-based Wii fit training in improving muscle strength, sensory integration ability, and walking abilities in patients with Parkinson's disease: a randomized control trial. International Journal of Gerontology, 9(4), pp.190-195.	Wrong outcomes
Liao, Y.Y., Chen, I.H., Lin, Y.J., Chen, Y. and Hsu, W.C., 2019. Effects of virtual reality-based physical and cognitive training on executive function and dual-task gait performance in older adults with mild cognitive impairment: a randomized control trial. Frontiers in aging neuroscience, 11, p.162.	Wrong outcomes
Lim, J., Cho, J.J., Kim, J., Kim, Y. and Yoon, B., 2017. Design of virtual reality training program for prevention of falling in the elderly: a pilot study on complex versus balance exercises. European Journal of Integrative Medicine, 15, pp.64-67.	Wrong outcomes
Liston, M., Genna, G., Maurer, C., Kikidis, D., Gatsios, D., Fotiadis, D., Bamiou, D.E. and Pavlou, M., 2021. Investigating the feasibility and acceptability of the HOLOBalance system compared with standard care in older adults at risk for falls: study protocol for an assessor blinded pilot randomised controlled study. BMJ open, 11(2), p.e039254.	Wrong publication type
Lupo, A., Giovanni Morone, M.D., Cinnera, A.M., Pucello, A., Coiro, P., Personeni, S., Francesca Gimigliano, M.D., Iolascon, G. and Paolucci, S., 2018. Effects on balance skills and patient compliance of biofeedback training with inertial measurement units and exergaming in subacute stroke: a pilot randomized controlled trial. Functional neurology, 33(3), pp.131-136.	Wrong population
Madhanraj Sekar et al. – The effectiveness of virtual reality (VR) therapy on balance and mobility in elderly patients: a randomized controlled trial – Fizjoterapia Polska 2024; 24(5); 191-194	Wrong publication type
Maidan, I., Rosenberg-Katz, K., Jacob, Y., Giladi, N., Hausdorff, J.M. and Mirelman, A., 2017. Disparate effects of training on brain activation in Parkinson disease. Neurology, 89(17), pp.1804-1810.	Wrong outcomes
Martel, D., Lauzé, M., Agnoux, A., de Laclos, L.F., Daoust, R., Émond, M., Sirois, M.J. and Aubertin-Leheudre, M., 2018. Comparing the effects of a home-based exercise program using a gerontechnology to a community-based group exercise program on functional capacities in older adults after a minor injury. Experimental gerontology, 108, pp.41-47.	Wrong outcomes
Mazzoleni, S., Montagnani, G., Vagheggini, G., Buono, L., Moretti, F., Dario, P. and Ambrosino, N., 2014. Interactive videogame as rehabilitation tool of	Wrong outcomes

patients with chronic respiratory diseases: preliminary results of a feasibility study. Respiratory medicine, 108(10), pp.1516-1524.	
Menengiç, K.N., Yeldan, İ., Cınar, N. and Şahiner, T.A., 2021. Effectiveness of home-based telerehabilitation in mild to moderate Alzheimer's disease: A randomised controlled study. Alzheimer's & Dementia, 17, p.e053406.	Wrong outcomes
Morat, M., Bakker, J., Hammes, V., Morat, T., Giannouli, E., Zijlstra, W. and Donath, L., 2019. Effects of stepping exergames under stable versus unstable conditions on balance and strength in healthy community-dwelling older adults: A three-armed randomized controlled trial. Experimental gerontology, 127, p.110719.	Wrong outcomes
Mora-Traverso, M., Molina-Garcia, P., Prieto-Moreno, R., Borges-Cosic, M., Cruz Guisado, V., del Pino Algarrada, R., Moreno-Ramírez, P., Gomez-Jurado, G., Gomez Tarrias, C., Hidalgo Isla, M. and Jimenez Andres, P., 2022. An m-Health telerehabilitation and health education program on physical performance in patients with hip fracture and their family caregivers: Study protocol for the ActiveHip+ randomized controlled trial. Research in Nursing & Health, 45(3), pp.287-299.	Wrong publication type
Moreira, N.B., Rodacki, A.L., Costa, S.N., Pitta, A. and Bento, P.C., 2021. Perceptive–cognitive and physical function in prefrail older adults: Exergaming versus traditional multicomponent training. Rejuvenation Research, 24(1), pp.28-36.	Wrong outcomes
Morone, G., Paolucci, T., Luziatelli, S., Iosa, M., Piermattei, C., Zangrando, F., Paolucci, S., Vulpiani, M.C., Saraceni, V.M., Baldari, C. and Guidetti, L., 2016. Wii Fit is effective in women with bone loss condition associated with balance disorders: a randomized controlled trial. Aging clinical and experimental research, 28(6), pp.1187-1193.	Wrong outcomes
Mugueta-Aguinaga, I. and Garcia-Zapirain, B., 2019. Frailty level monitoring and analysis after a pilot six-week randomized controlled clinical trial using the FRED exergame including biofeedback supervision in an elderly day care centre. International journal of environmental research and public health, 16(5), p.729.	Wrong outcomes
Oesch, P., Kool, J., Fernandez-Luque, L., Brox, E., Evertsen, G., Civit, A., Hilfiker, R. and Bachmann, S., 2017. Exergames versus self-regulated exercises with instruction leaflets to improve adherence during geriatric rehabilitation: a randomized controlled trial. BMC geriatrics, 17(1), p.77.	Wrong outcomes
Padala, K.P., Padala, P., Lensing, S.Y., Dennis, R.A., Bopp, M.M., Garner, K.K., Parkes, C.M., Roberson, P., Dubbert, P.M. and Sullivan, D., 2016, May. Do exercises performed by exergames improve balance in older adults?. In JOURNAL OF THE AMERICAN GERIATRICS SOCIETY (Vol. 64, pp. S167-S167). 111 RIVER ST, HOBOKEN 07030-5774, NJ USA: WILEY-BLACKWELL.	Wrong outcomes
Padala, K.P., Padala, P.R., Lensing, S.Y., Dennis, R.A., Bopp, M.M., Parkes, C.M., Garrison, M.K., Dubbert, P.M., Roberson, P.K. and Sullivan, D.H., 2017. Efficacy of wii-fit on static and dynamic balance in community dwelling older	Wrong outcomes

veterans: a randomized controlled pilot trial. Journal of aging research, 2017(1), p.4653635.	
Padala, K.P., Padala, P.R., Lensing, S.Y., Dennis, R.A., Bopp, M.M., Roberson, P.K. and Sullivan, D.H., 2017. Home-based exercise program improves balance and fear of falling in community-dwelling older adults with mild Alzheimer's disease: a pilot study. Journal of Alzheimer's disease, 59(2), pp.565-574.	Wrong outcomes
Padala, K.P., Malloy, T.R., Lensing, S.Y., Bopp, M.M., Sullivan, D.H. and Padala, P.R., 2019. P4-665: EXERCISE ADHERENCE IN EARLY ALZHEIMER'S DEMENTIA: WHAT ROLES DO EXECUTIVE FUNCTION AND APATHY PLAY?. Alzheimer's & Dementia, 15, pp.P1586-P1586.	Wrong outcomes
Park, J.H. and Park, J.H., 2018. Does cognition-specific computer training have better clinical outcomes than non-specific computer training? A single-blind, randomized controlled trial. Clinical rehabilitation, 32(2), pp.213-222.	Wrong outcomes
Pellegrini, C.A., Lee, J., DeVivo, K.E., Harpine, C.E., Del Gaizo, D.J. and Wilcox, S., 2021. Reducing sedentary time using an innovative mHealth intervention among patients with total knee replacement: Rationale and study protocol. Contemporary clinical trials Communications, 22, p.100810.	Wrong outcomes
Piñero, D.P., Molina-Martin, A., Ramón, M.L., Rincón, J.L., Fernández, C., de Fez, D., Arenillas, J.F., Leal-Vega, L., Coco-Martín, M.B. and Maldonado, M.J., 2021. Preliminary evaluation of the clinical benefit of a novel visual rehabilitation program in patients implanted with trifocal diffractive intraocular lenses: a blinded randomized placebo-controlled clinical trial. Brain Sciences, 11(9), p.1181.	Wrong outcomes
Pluchino, A., Lee, S.Y., Asfour, S., Roos, B.A. and Signorile, J.F., 2012. Pilot study comparing changes in postural control after training using a video game balance board program and 2 standard activity-based balance intervention programs. Archives of physical medicine and rehabilitation, 93(7), pp.1138-1146.	Wrong outcomes
Rendon, A.A., 2011. Virtual reality gaming as a tool for rehabilitation in physical therapy. Loma Linda University.	Wrong outcomes
Rendon, A.A., Lohman, E.B., Thorpe, D., Johnson, E.G., Medina, E. and Bradley, B., 2012. The effect of virtual reality gaming on dynamic balance in older adults. Age and ageing, 41(4), pp.549-552.	Wrong outcomes
Rhodus, E.K., Baum, C., Kryscio, R., Liu, C., George, R., Thompson, M., Lowry, K., Coy, B., Barber, J., Nichols, H. and Curtis, A., 2023. Feasibility of telehealth occupational therapy for behavioral symptoms of adults with dementia: randomized controlled trial. The American Journal of Occupational Therapy, 77(4), p.7704205010.	Wrong outcomes
Rica, R.L., Shimojo, G.L., Gomes, M.C., Alonso, A.C., Pitta, R.M., Santa-Rosa, F.A., Pontes Junior, F.L., Ceschini, F., Gobbo, S., Bergamin, M. and Bocalini, D.S., 2020. Effects of a Kinect-based physical training program on body	Wrong outcomes

composition, functional fitness and depression in institutionalized older adults. Geriatrics & gerontology international, 20(3), pp.195-200.	
Sadura-Sieklucka, T., Czerwosz, L.T., Kądalska, E., Kożuchowski, M., Księżopolska-Orłowska, K. and Targowski, T., 2023. Is balance training using biofeedback effective in the prophylaxis of falls in women over the age of 65?. Brain sciences, 13(4), p.629.	Wrong intervention
Salgueiro, C., Urrútia, G. and Cabanas-Valdés, R., 2022. Telerehabilitation for balance rehabilitation in the subacute stage of stroke: A pilot controlled trial. NeuroRehabilitation, 51(1), pp.91-99.	Wrong intervention
Sato, K., Kuroki, K., Saiki, S. and Nagatomi, R., 2015. Improving walking, muscle strength, and balance in the elderly with an exergame using Kinect: A randomized controlled trial. Games for health journal, 4(3), pp.161-167.	Wrong outcomes
Scanlon A-M, Belton A, Magnier A, Coleman K, O'Neill D, Meldrum D. Use of Nintendo Wii® and its effect on the balance of older adults at risk of falls: a pilot randomised controlled trial. European geriatric medicine 2010; 1: S156.	Wrong outcomes
Schättin, A., Baier, C., Mai, D., Klamroth-Marganska, V., Herter-Aeberli, I. and de Bruin, E.D., 2019. Effects of exergame training combined with omega-3 fatty acids on the elderly brain: a randomized double-blind placebo-controlled trial. BMC geriatrics, 19(1), p.81.	Wrong outcomes
Scherrenberg, M., Zeymer, U., Schneider, S., Van der Velde, A.E., Wilhelm, M., Van't Hof, A.W.J., Kolkman, E., Prins, L.F., Prescott, E., Iliou, M.C. and Peña-Gil, C., 2021. EU-CaRE study: could exercise-based cardiac telerehabilitation also be cost-effective in elderly?. International journal of cardiology, 340, pp.1-6.	Wrong outcomes
Schoene, D., Lord, S.R., Delbaere, K., Severino, C., Davies, T.A. and Smith, S.T., 2013. A randomized controlled pilot study of home-based step training in older people using videogame technology. PloS one, 8(3), p.e57734.	Wrong outcomes
Schumacher, H., Stüwe, S., Kropp, P., Diedrich, D., Freitag, S., Greger, N., Junghanss, C., Freund, M. and Hilgendorf, I., 2018. A prospective, randomized evaluation of the feasibility of exergaming on patients undergoing hematopoietic stem cell transplantation. Bone Marrow Transplantation, 53(5), pp.584-590.	Wrong outcomes
Schwenk, M., Grewal, G.S., Honarvar, B., Schwenk, S., Mohler, J., Khalsa, D.S. and Najafi, B., 2014. Interactive balance training integrating sensor-based visual feedback of movement performance: a pilot study in older adults. Journal of neuroengineering and rehabilitation, 11(1), p.164.	Wrong outcomes
Schwenk, M., Sabbagh, M., Lin, I., Morgan, P., Grewal, G.S., Mohler, J., Coon, D.W. and Najafi, B., 2016. Sensor-based balance training with motion feedback in people with mild cognitive impairment. Journal of rehabilitation research and development, 53(6), p.945.	Wrong outcomes
Schwenk, M., Grewal, G.S., Holloway, D., Muchna, A., Garland, L. and Najafi,	Wrong outcomes

B., 2016. Interactive sensor-based balance training in older cancer patients with chemotherapy-induced peripheral neuropathy: a randomized controlled trial. Gerontology, 62(5), pp.553-563.	
Segura-Orti, E., Martínez-Olmos, F.J., Ortega-Perez, L., Gómez-Conesa, A., Amer-Cuenca, J.J., Valtueña-Gimeno, N., Meléndez-Oliva, E., Martínez-Gramage, J., García-Testal, A., Montañez-Aguilera, J. and Arguisuelas-Martínez, D., 2019. SP421 VIRTUAL REALITY EXERCISE DURING HEMODIALYSIS TO IMPROVE HEALTH RELATED QUALITY OF LIFE: RANDOMIZED CONTROLLED TRIAL. Nephrology Dialysis Transplantation, 34(Supplement_1), pp.gfz103-SP421.	Wrong outcomes
Segura-Ortí, E., Martínez-Olmos, F.J., Garcia-Testal, A., Garcia-Maset, R., Valtueña-Gimeno, N., Ortega-Perez de Villar, L., Piñón-Ruiz, J., Ferrer-Salva, A., Karandysz, K., Lehtoviita, M.E.A. and Ferrer-Sargues, F.J., 2020, June. VIRTUAL REALITY EXERCISE INTRADIALYSIS IMPACT ON PHYSICAL FUNCTION AND PHYSICAL ACTIVITY LEVEL: RANDOMIZED CONTROLLED TRIAL WITH FOLLOW-UP. In Nephrology Dialysis Transplantation (Vol. 35, No. Supplement_3).	Wrong outcomes
Shake, M.C., Crandall, K.J., Mathews, R.P., Falls, D.G. and Dispennette, A.K., 2018. Efficacy of Bingocize®: A game-centered mobile application to improve physical and cognitive performance in older adults. Games for health journal, 7(4), pp.253-261.	Wrong outcomes
Signorile, J.F., Pluchino, A., Lee, S.Y., Asfour, S.S. and Roos, B.A., 2011, April. Wii Fit Balance Produces Similar Improvements in Balance and Postural Control to Formalized Training. In Journal of the American Geriatrics Society (Vol. 59, pp. S18-S18). COMMERCE PLACE, 350 MAIN ST, MALDEN 02148, MA USA: WILEY-BLACKWELL.	Wrong outcomes
Singh, D.K., Rajaratnam, B.S., Palaniswamy, V., Pearson, H., Raman, V.P. and Bong, P.S., 2012. Participating in a virtual reality balance exercise program can reduce risk and fear of falls. Maturitas, 73(3), pp.239-243.	Wrong outcomes
Slegers, K., Van Boxtel, M.P. and Jolles, J., 2008. Effects of computer training and Internet usage on the well-being and quality of life of older adults: a randomized, controlled study. The journals of gerontology series B: Psychological sciences and social sciences, 63(3), pp.P176-P184.	Wrong outcomes
Smaerup, M., Grönvall, E., Larsen, S.B., Laessoe, U., Henriksen, J.J. and Damsgaard, E.M., 2015. Computer-assisted training as a complement in rehabilitation of patients with chronic vestibular dizziness—a randomized controlled trial. Archives of physical medicine and rehabilitation, 96(3), pp.395-401.	Wrong outcomes
Smith-Ray, R.L., Hughes, S.L., Prohaska, T.R., Little, D.M., Jurivich, D.A. and Hedeker, D., 2015. Impact of cognitive training on balance and gait in older adults. Journals of Gerontology Series B: Psychological Sciences and Social Sciences, 70(3), pp.357-366.	Wrong outcomes
Sörlén, N., Hult, A., Nordström, P., Nordström, A. and Johansson, J., 2021.	Wrong

Short-term balance training and acute effects on postural sway in balance-deficient older adults: a randomized controlled trial. BMC Sports Science, Medicine and Rehabilitation, 13(1), p.23.	intervention
Soto-Varela, A., Rossi-Izquierdo, M., del-Río-Valeiras, M., Faraldo-García, A., Vaamonde-Sánchez-Andrade, I., Lirola-Delgado, A. and Santos-Pérez, S., 2021. Vestibular rehabilitation with mobile posturography as a “low-cost” alternative to vestibular rehabilitation with computerized dynamic posturography, in old people with imbalance: a randomized clinical trial. Aging Clinical and Experimental Research, 33(10), pp.2807-2819.	Wrong outcomes
Tallner, A., Streber, R., Hentschke, C., Morgott, M., Geidl, W., Mäurer, M. and Pfeifer, K., 2016. Internet-supported physical exercise training for persons with multiple sclerosis—a randomised, controlled study. International journal of molecular sciences, 17(10), p.1667.	Wrong outcomes
Tallon, G., Seilles, A., Mélia, G., Andary, S., Bernard, P., Di Loreto, I. and Blain, H., 2015. Effects of the serious game Medimoov on the functional autonomy of institutionalized older adults. Annals of Physical and Rehabilitation Medicine, 58, pp.e113-e114.	Wrong outcomes
Taylor, L., Kerse, N., Klenk, J., Borotkanics, R. and Maddison, R., 2018. Exergames to improve the mobility of long-term care residents: a cluster randomized controlled trial. Games for health journal, 7(1), pp.37-42.	Wrong outcomes
Tollar, J., Nagy, F., Moizs, M., Toth, B.E., Sanders, L.M. and Hortobagyi, T., 2019. Diverse exercises similarly reduce older adults' mobility limitations. Medicine and science in sports and exercise, 51(9), pp.1809-1816.	Wrong outcomes
Tollár, J., Nagy, F. and Hortobágyi, T., 2019. Vastly different exercise programs similarly improve parkinsonian symptoms: a randomized clinical trial. Gerontology, 65(2), pp.120-127.	Wrong outcomes
Toquero, M., Caballer, V.B., Martínez-Olmos, F.J., Valtueña-Gimeno, N., Garcia-Testal, A., Cana-Poyatos, A., Rico-Salvador, I., Narbona, V.V., Gil-Gómez, J.A. and Segura, E., 2023. # 4416 MAINTAINING PHYSICAL ACTIVITY IN PATIENTS THROUGH INTRADIALYTIC VIRTUAL REALITY EXERCISE INTERVENTION: THE REVID STUDY. Nephrology Dialysis Transplantation, 38(Supplement_1), p.gfad063c_4416.	Wrong publication type
Trinh, L., Arbour-Nicitopoulos, K.P., Sabiston, C.M., Berry, S.R., Loblaw, A., Alibhai, S.M., Jones, J.M. and Faulkner, G.E., 2018. RiseTx: testing the feasibility of a web application for reducing sedentary behavior among prostate cancer survivors receiving androgen deprivation therapy. International Journal of Behavioral Nutrition and Physical Activity, 15(1), p.49.	Wrong study design
Trippo, K., Ferraz, D., Farinha, K., Kruschewsky, R. and Oliveira-Filho, J., 2017, February. Postural balance and elderly quality of life with Parkinson's disease treated with functional training, stationary bike and exergame: pilot study of a randomized clinical trial. In Movement Disorders (Vol. 32, pp. S41-S42). 111 RIVER ST, HOBOKEN 07030-5774, NJ USA: WILEY.	Wrong outcomes

Tsang, W.W.N., Fong, S.S.M., Tung, K.K. and Fu, A.S.N., 2015. Is virtual reality exercise effective in reducing falls among older adults with a history of falls?. Physiotherapy, 101, pp.e1539-e1540.	Wrong publication type
Tuan, S.H., Chang, L.H., Sun, S.F., Li, C.H., Chen, G.B. and Tsai, Y.J., 2024. Assessing the clinical effectiveness of an exergame-based exercise training program using ring fit adventure to prevent and postpone frailty and sarcopenia among older adults in rural long-term care facilities: Randomized controlled trial. Journal of medical Internet research, 26, p.e59468.	Wrong outcomes
Turunen, K.M., Tirkkonen, A., Savikangas, T., Hänninen, T., Alen, M., Fielding, R.A., Kivipelto, M., Stigsdotter Neely, A., Törmäkangas, T. and Sipilä, S., 2022. Effects of physical and cognitive training on falls and concern about falling in older adults: results from a randomized controlled trial. The Journals of Gerontology: Series A, 77(7), pp.1430-1437.	Wrong intervention
Uğur, F. and Sertel, M., 2020. The effect of virtual reality applications on balance and gait speed in individuals with Alzheimer dementia: a pilot study. Topics in Geriatric Rehabilitation, 36(4), pp.221-229.	Wrong outcomes
van den Berg, M., Sherrington, C., Killington, M., Smith, S., Bongers, B., Hassett, L. and Crotty, M., 2016. Video and computer-based interactive exercises are safe and improve task-specific balance in geriatric and neurological rehabilitation: a randomised trial. Journal of physiotherapy, 62(1), pp.20-28.	Wrong outcomes
Van Der Weegen, S., Verwey, R., Spreeuwenberg, M., Tange, H., van der Weijden, T. and de Witte, L., 2015. It's LiFe! Mobile and web-based monitoring and feedback tool embedded in primary care increases physical activity: a cluster randomized controlled trial. Journal of medical Internet research, 17(7), p.e4579.	Wrong population
Van Schooten, K.S., Callisaya, M.L., O" Dea, B., Lung, T., Anstey, K., Lord, S.R., Christensen, H., Brown, A., Chow, J., McInerney, G. and Miles, L., 2021. Protocol of a 12-month multifactorial eHealth programme targeting balance, dual-tasking and mood to prevent falls in older people: the StandingTall+ randomised controlled trial. BMJ open, 11(4), p.e051085.	Wrong publication type
Villumsen, B.R., Jorgensen, M.G., Frystyk, J., Hørdam, B. and Borre, M., 2019. Home-based 'exergaming' was safe and significantly improved 6-min walking distance in patients with prostate cancer: a single-blinded randomised controlled trial. BJU international, 124(4), pp.600-608.	Wrong outcomes
Vorrink, S.N., Kort, H.S., Troosters, T., Zanen, P. and Lammers, J.W.J., 2016. Efficacy of an mHealth intervention to stimulate physical activity in COPD patients after pulmonary rehabilitation. European Respiratory Journal, 48(4), pp.1019-1029.	Wrong outcomes
Wang, L., Guo, Y., Wang, M. and Zhao, Y., 2021. A mobile health application to support self-management in patients with chronic obstructive pulmonary disease: a randomised controlled trial. Clinical rehabilitation, 35(1), pp.90-	Wrong outcomes

101.	
Whyatt, C., Merriman, N.A., Young, W.R., Newell, F.N. and Craig, C., 2015. A Wii bit of fun: a novel platform to deliver effective balance training to older adults. Games for health journal, 4(6), pp.423-433.	Wrong outcomes
Wolf, S.L., Barnhart, H.X., Kutner, N.G., McNeely, E., Coogler, C., Xu, T. and Atlanta FICSIT Group, 1996. Reducing frailty and falls in older persons: an investigation of Tai Chi and computerized balance training. Journal of the American Geriatrics Society, 44(5), pp.489-497.	Wrong intervention
Wu, Z., Xu, J., Yue, C., Li, Y. and Liang, Y., 2020. Collaborative care model based telerehabilitation exercise training program for acute stroke patients in China: a randomized controlled trial. Journal of Stroke and Cerebrovascular Diseases, 29(12), p.105328.	Wrong population
Wu, S., Ji, H., Won, J., Jo, E.A., Kim, Y.S. and Park, J.J., 2023. The effects of exergaming on executive and physical functions in older adults with dementia: randomized controlled trial. Journal of medical Internet research, 25, p.e39993.	Wrong outcomes
Yaqoob, I. and Khan, S.U., 2018. Effectiveness of balance training on quality of life in osteoporotic women. Rawal Medical Journal, 43(2), pp.328-328.	Wrong outcomes
Yalfani, A., Abedi, M. and Raeisi, Z., 2022. Effects of an 8-week virtual reality training program on pain, fall risk, and quality of life in elderly women with chronic low back pain: Double-blind randomized clinical trial. Games for Health Journal, 11(2), pp.85-92.	Wrong outcomes
Yang, W.C., Wang, H.K., Wu, R.M., Lo, C.S. and Lin, K.H., 2016. Home-based virtual reality balance training and conventional balance training in Parkinson's disease: A randomized controlled trial. Journal of the Formosan Medical Association, 115(9), pp.734-743.	Wrong outcomes
Yang, C.M., Hsieh, J.S.C., Chen, Y.C., Yang, S.Y. and Lin, H.C.K., 2020. Effects of Kinect exergames on balance training among community older adults: A randomized controlled trial. Medicine, 99(28), p.e21228.	Wrong outcomes
Yerlikaya, T., Öniz, A. and Özgùren, M., 2021. The effect of an interactive tele rehabilitation program on balance in older individuals. Neurological Sciences and Neurophysiology, 38(3), pp.180-186.	Wrong outcomes
Yeşilyaprak, S.S., Yıldırım, M.Ş., Tomruk, M., Ertekin, Ö. and Algun, Z.C., 2016. Comparison of the effects of virtual reality-based balance exercises and conventional exercises on balance and fall risk in older adults living in nursing homes in Turkey. Physiotherapy theory and practice, 32(3), pp.191-201.	Wrong outcomes
Yuan, R.Y., Chen, S.C., Peng, C.W., Lin, Y.N., Chang, Y.T. and Lai, C.H., 2020. Effects of interactive video-game–based exercise on balance in older adults with mild-to-moderate Parkinson’s disease. Journal of neuroengineering and rehabilitation, 17(1), p.91.	Wrong outcomes

Yuen, H.K., Lowman, J.D., Oster, R.A. and de Andrade, J.A., 2019. Home-based pulmonary rehabilitation for patients with idiopathic pulmonary fibrosis: a pilot study. Journal of cardiopulmonary rehabilitation and prevention, 39(4), pp.281-284.	Wrong outcomes
Zadro, J.R., Shirley, D., Simic, M., Mousavi, S.J., Ceprnja, D., Maka, K., Sung, J. and Ferreira, P., 2019. Video-game–based exercises for older people with chronic low back pain: a randomized controlledtable trial (GAMEBACK). Physical therapy, 99(1), pp.14-27.	Wrong outcomes
Zadro, J.R., Shirley, D., Nilsen, T.I., Mork, P.J. and Ferreira, P.H., 2020. Family history influences the effectiveness of home exercise in older people with chronic low back pain: a secondary analysis of a randomized controlled trial. Archives of physical medicine and rehabilitation, 101(8), pp.1322-1331.	Wrong outcomes

Reference list of studies excluded during backward citation search on included reviews

de Bruin ED, Reith A, Dörflinger M, Murer K. Feasibility of strength balance training extended with computer game dancing in older people; does it affect dual task costs of walking? J Nov Physiother. 2011; 1:104.	Wrong outcomes
de Bruin ED, Reith A, Dörflinger M, Murer K. Feasibility of strength balance training extended with computer game dancing in older people; does it affect dual task costs of walking? J Nov Physiother. 2011; 1:104.	Wrong outcomes
Eggenberger P, Schumacher V, Angst M, Theill N, de Bruin ED. Does multicomponent physical exercise with simultaneous cognitive training boost cognitive performance in older adults? A 6-month randomized controlled trial with a 1-year follow-up. Clin Interv Aging. 2015;10:1335–1349.	Wrong outcomes
Eggenberger P, Schumacher V, Angst M, Theill N, de Bruin ED. Does multicomponent physical exercise with simultaneous cognitive training boost cognitive performance in older adults? A 6-month randomized controlled trial with a 1-year follow-up. Clin Interv Aging. 2015;10:1335–1349.	Duplicate
Ferraz DD, Trippo KV, Duarte GP, Neto MG, Bernardes Santos KO, Filho JO. The effects of functional training, bicycle exercise, and exergaming on walking capacity of elderly patients with Parkinson disease: a pilot randomized controlled single-blinded trial. Arch Phys Med Rehab 2018; 99: 826–33	Wrong outcomes
Fu AS, Gao KL, Tung AK, Tsang WW, Kwan MM. Eff ectiveness of exergaming training in reducing risk and incidence of falls in frail older adults with a history of falls. Arch Phys Med Rehabil 2015; 96: 2096–102.	Duplicate
Gomes, G.C.V., do Socorro Simões, M., Lin, S.M., Bacha, J.M.R., Viveiro, L.A.P., Varise, E.M., Junior, N.C., Lange, B., Jacob Filho, W. and Pompeu, J.E., 2018. Feasibility, safety, acceptability, and functional outcomes of playing Nintendo Wii Fit PlusTM for frail older adults: A randomized feasibility clinical trial. Maturitas, 118, pp.20-28.	Wrong outcomes
Griffin, M., McCormick, D., Taylor, M.J., Shawis, T. and Impson, R., 2012. Using the Nintendo Wii as an intervention in a falls prevention group.	Wrong study design
Gudlaugsson J, Gudnason V, Aspelund T, et al. Effects of a 6-month multimodal training intervention on retention of functional fitness in older adults: a randomized-controlled cross-over design. Int J Behav Nutr Phys Act. 2012;9:107.	Wrong outcomes
Gudlaugsson J, Gudnason V, Aspelund T, et al. Effects of a 6-month multimodal training intervention on retention of functional fitness in older adults: a randomized-controlled cross-over design. Int J Behav Nutr Phys Act. 2012;9:107.	Wrong outcomes

Hoang P, Schoene D, Gandevia S, et al. Effects of a homebased step training programme on balance, stepping, cognition and functional performance in people with multiple sclerosis—a randomized controlled trial. Mult Scler 2016; 22: 94–103	Wrong outcomes
J. E. Pompeu, F.A.D.S.Mendes, K.G. D. Silvaetal.,"Effect of Nintendo Wii-based motor and cognitive training on activities ofdaily living in patients with Parkinson's disease: a randomised clinical trial," Physiotherapy, vol. 98, no. 3, pp. 196–204, 2012.	Wrong outcomes
J. E. Pompeu,F.A.D.S.Mendes, K.G. D. Silvaetal.,"Effect of Nintendo Wii-based motor and cognitive training on activities ofdaily living in patients with Parkinson's disease: a randomised clinical trial," Physiotherapy, vol. 98, no. 3, pp. 196–204, 2012.	Wrong outcomes
J.-F. Esculier, J. Vaudrin, P. B´eriault, K. Gagnon, and L. E. Tremblay, "Home-based balance training programme using Wii Fit with balance board for Parkinsons's disease: a pilot study," Journal of Rehabilitation Medicine,vol.44,no.2,pp.144–150, 2012.	Wrong outcomes
J.-F. Esculier, J. Vaudrin, P. B´eriault, K. Gagnon, and L. E. Tremblay, "Home-based balance training programme using Wii Fit with balance board for Parkinsons's disease: a pilot study," Journal of Rehabilitation Medicine,vol.44,no.2,pp.144–150, 2012.	Wrong publication
Jorgensen, Martin G., Uffe Laessoe, Carsten Hendriksen, Ole Bruno Faurholt Nielsen, and Per Aagaard. "Efficacy of Nintendo Wii training on mechanical leg muscle function and postural balance in community-dwelling older adults: a randomized controlled trial." Journals of Gerontology Series A: Biomedical Sciences and Medical Sciences 68, no. 7 (2013): 845-852.	Wrong outcomes
Lloréns, R., Gil-Gómez, J.A., Alcañiz, M., Colomer, C. and Noé, E., 2015. Improvement in balance using a virtual reality-based stepping exercise: a randomized controlled trial involving individuals with chronic stroke. Clinical rehabilitation, 29(3), pp.261-268.	Wrong outcomes
Lloréns, Roberto, Enrique Noé, Carolina Colomer, and Mariano Alcañiz. "Effectiveness, usability, and cost-benefit of a virtual reality–based telerehabilitation program for balance recovery after stroke: A randomized controlled trial." Archives of physical medicine and rehabilitation 96, no. 3 (2015): 418-425.	Duplicate
Mirelman A, Rochester L, Maidan I, et al. Addition of a non-immersive virtual reality component to treadmill training to reduce fall risk in older adults (V-TIME): a randomised controlled trial. Lancet 2016; 188: 1170–1182	Duplicate
Mirelman A, Rochester L, Reelick M, et al. V-TIME: a treadmill training program augmented by virtual reality to decrease fall risk in older adults: study design of a randomized controlled trial. BMC Neurol 2013; 13: 15	Wrong publication
Mirelman A, Rochester L, Reelick M, et al. V-TIME: a treadmill training program augmented by virtual reality to decrease fall risk in older adults:	Wrong

study design of a randomized controlled trial. BMC Neurol 2013; 13: 15.	publication
Mirelman A, Rochester L, Maidan I, et al. Addition of a non-immersive virtual reality component to treadmill training to reduce fall risk in older adults (V-TIME): a randomised controlled trial. Lancet. 2016;388:11701182. doi: 10.1016/S0140-6736(16)31325-3	Duplicate
Mirelman A, Rochester L, Maidan I, et al. Addition of a non-immersive virtual reality component to treadmill training to reduce fall risk in older adults (V-TIME): a randomised controlled trial. Lancet. 2016;388:11701182. doi: 10.1016/S0140-6736(16)31325-3	Duplicate
Mirelman A, Rochester L, Maidan I, et al. Addition of a non-immersive virtual reality component to treadmill training to reduce fall risk in older adults (V-TIME): a randomised controlled trial. Lancet. 2016;388:11701182. doi: 10.1016/S0140-6736(16)31325-3	Duplicate
Mirelman A, Rochester L, Maidan I, et al. Addition of a non-immersive virtual reality component to treadmill training to reduce fall risk in older adults (V-TIME): a randomised controlled trial. Lancet. 2016;388:11701182. doi: 10.1016/S0140-6736(16)31325-3	Duplicate
Mirelman A, Rochester L, Reelick M, et al. V-TIME: a treadmill training program augmented by virtual reality to decrease fall risk in older adults: study design of a randomized controlled trial. BMC Neurol. 2013;13:15. doi: 10.1186/1471-2377-13-15	Duplicate
Mirelman A, Rochester L, Reelick M, et al. V-TIME: a treadmill training program augmented by virtual reality to decrease fall risk in older adults: study design of a randomized controlled trial. BMC Neurol. 2013;13:15. doi: 10.1186/1471-2377-13-15	Duplicate
Pichierri G, Murer K, de Bruin ED. A cognitive-motor intervention using a dance video game to enhance foot placement accuracy and gait under dual task conditions in	Wrong outcomes
Pichierri G, Murer K, de Bruin ED. A cognitive-motor intervention using a dance video game to enhance foot placement accuracy and gait under dual task conditions in	Wrong outcomes
Pompeu, J.E., Andrade, G., Mendonça, M.S., Pompeu, S.M.A.A. and Lange, B., 2014. Safety, feasibility and effectiveness of balance and gait training using Nintendo Wii Fit Plus on unstable surface in patients with Parkinson’s disease: a pilot study. J Alzheimers Dis Parkinsonism, 4(1), pp.1-4.	Wrong outcomes
Pompeu, J.E., dos Santos Mendes, F.A., da Silva, K.G., Lobo, A.M., de Paula Oliveira, T., Zomignani, A.P. and Piemonte, M.E.P., 2012. Effect of Nintendo Wii™-based motor and cognitive training on activities of daily living in patients with Parkinson's disease: a randomised clinical trial. Physiotherapy, 98(3), pp.196-204.	Duplicate

R. Llor´ens, E. No´e, C. Colomer, and M. Alca˜niz, “Effectiveness, usability, and cost-benefit of a virtual reality-based telerehabilitation program for balance recovery after stroke: a randomized controlled trial,” Archives of Physical Medicine and Rehabilitation,vol.96, no.3,pp. 418–425.e2,2015.	Wrong outcomes
R. Llor´ens, E. No´e, C. Colomer, and M. Alca˜niz, “Effectiveness, usability, and cost-benefit of a virtual reality-based telerehabilitation program for balance recovery after stroke: a randomized controlled trial,” Archives of Physical Medicine and Rehabilitation,vol.96, no.3,pp. 418–425.e2,2015.	Wrong outcomes
Schenkman M, Hall DA, Baron AE, Schwartz RS, Mettler P, Kohrt WM. Exercise for people in early- or mid-stage Parkinson disease: a 16-month randomized controlled trial. Phys Ther 2012; 92: 1395–410	Wrong intervention
Schoene D, Lord SR, Delbaere K, et al. A randomized controlled pilot study of home-based step training in older people using videogame technology. PLoS ONE 2013; 8: e57734	Wrong outcomes
Song 2018. Home-based step training using videogame technology in people with Parkinson’s disease: a single-blinded randomised controlled trial. Clin. Rehabil. 32, 299–311 (2018).	Duplicate
Song, J. et al. Home-based step training using videogame technology in people with Parkinson’s disease: a single-blinded randomised controlled trial. Clin. Rehabil. 32, 299–311 (2018).	Duplicate
Song, J. et al. Home-based step training using videogame technology in people with Parkinson’s disease: a single-blinded randomised controlled trial. Clin. Rehabil. 32, 299–311 (2018).	Duplicate
Stanmore EK, Mavroeidi A, de Jong LD, Skelton DA, Sutton CJ, Benedetto V, et al. The effectiveness and cost-effectiveness of strength and balance Exergames to reduce falls risk for people aged 55 years and older in UK assisted living facilities: a multi-centre, cluster randomised controlled trial. BMC Med. 2019 Feb 28;17(1):49. PMID: 30813926. doi: 10.1186/s12916-019-1278-9.	Duplicate
Stanmore E, Mavroeidi A, Meekes W, Skelton D, Sutton C, Benedetto V, de Jong LD, Todd C. Exergames to reduce falls risk in older people in UK assisted living facilities: A multi-centre cluster RCT. Innovation in Aging. 2018;2(Suppl 1):362–63. https://doi.org/10.1093/geroni/igy023.1340.	Wrong publication
Sturnieks, D. L. et al. Efect of cognitive-only and cognitive-motor training on preventing falls in community-dwelling older people: protocol for the smart±step randomised controlled trial. BMJ Open 9, e029409 (2019)	Wrong publication
Van der Kolk NM, de Vries NM, Kessels RP, Joosten H, Zwinderman AH, Post B, Bloem BR. Effectiveness of home-based and remotely supervised aerobic exercise in Parkinson's disease: a double-blind, randomised controlled trial. The Lancet Neurology. 2019 Nov 1;18(11):998-1008.	Wrong outcomes

van der Kolk NM, de Vries NM, Penko AL, et al. A remotely supervised home-based aerobic exercise programme is feasible for patients with Parkinson's disease: results of a small randomised feasibility trial. J Neurol Neurosurg Psychiatry 2017; 89(9): 1003–5.	Wrong outcomes

Reference list of studies excluded during forward Citation Search on included reviews

Begde, A., Alqurafi, A., Pain, M.T., Blenkinsop, G., Wilcockson, T.D. and Hogervorst, E., 2023. The effectiveness of home-based exergames training on cognition and balance in older adults: a comparative quasi-randomized study of two exergame interventions. Innovation in Aging, 7(8), p.igad102.	Wrong outcome
Canning, C.G., Allen, N.E., Nackaerts, E., Paul, S.S., Nieuwboer, A. and Gilat, M., 2020. Virtual reality in research and rehabilitation of gait and balance in Parkinson disease. *Nature Reviews Neurology*, *16*(8), pp.409-425.	Wrong outcomes
Chen, X., Wu, L., Feng, H., Ning, H., Wu, S., Hu, M., Jiang, D., Chen, Y., Jiang, Y. and Liu, X., 2023. Comparison of exergames versus conventional exercises on the health benefits of older adults: Systematic review with meta-analysis of randomized controlled trials. JMIR Serious Games, 11, p.e42374.	Wrong outcome
Chen, Y., Zhang, Y., Guo, Z., Bao, D. and Zhou, J., 2021. Comparison between the effects of exergame intervention and traditional physical training on improving balance and fall prevention in healthy older adults: a systematic review and meta-analysis. Journal of neuroengineering and rehabilitation, 18(1), p.164.	Wrong outcome
Galperin, I., Mirelman, A., Schmitz-Hübsch, T., Hsieh, K.L., Regev, K., Karni, A., Brozgol, M., Cornejo Thumm, P., Lynch, S.G., Paul, F. and Devos, H., 2023. Treadmill training with virtual reality to enhance gait and cognitive function among people with multiple sclerosis: a randomized controlled trial. Journal of Neurology, 270(3), pp.1388-1401.	Wrong population
Garcia-Lopez, H., Obrero-Gaitan, E., Castro-Sanchez, A.M., Lara-Palomo, I.C., Nieto-Escamez, F.A. and Cortes-Perez, I., 2021. Non-immersive virtual reality to improve balance and reduce risk of falls in people diagnosed with parkinson's disease: a systematic review. Brain sciences, 11(11), p.1435.	Wrong outcome
Gorgey, A.S., Goldsmith, J.A., Anderson, M. and Castillo, T., 2022. Telerehabilitation for Exercise in Neurological Disability. In Telerehabilitation (pp. 319-337). Elsevier.	Wrong publication
Hicks, C., Smith, N., Ratanapongleka, M., Menant, J., Turner, J., Lo, J., Garcia, J., Valenzuela, M., Chaplin, C., Delbaere, K. and Herber, R., 2023. smart±step exergame and seated computer brain training for preventing falls in community-dwelling older people: a 12-month randomised controlled trial.	Duplicate
Kwok, B.C. and Pua, Y.H., 2016. Effects of WiiActive exercises on fear of falling and functional outcomes in community-dwelling older adults: a randomised control trial. Age and ageing, 45(5), pp.621-627.	Duplicate
Lapierre, N., Din, N.U., Igout, M., Chevrier, J. and Belmin, J., 2021. Effects of a rehabilitation program using a patient-personalized exergame on fear of falling and risk of falls in vulnerable older adults: protocol for a randomized	Wrong outcome

controlled group study. JMIR research protocols, 10(8), p.e24665.	
Leavy, B., Sedhed, J., Kalbe, E., Åkesson, E., Franzén, E. and Johansson, H., 2023. Design of the STEPS trial: a phase II randomized controlled trial evaluating eHealth-supported motor-cognitive home training for Parkinson's disease. BMC neurology, 23(1), p.356.	Wrong publication
Li, A., Li, J., Zhang, D., Wu, W., Zhao, J. and Qiang, Y., 2023. Synergy through integration of digital cognitive tests and wearable devices for mild cognitive impairment screening. Frontiers in Human Neuroscience, 17, p.1183457.	Wrong outcome
Liao, Y.Y., Chen, I.H., Lin, Y.J., Chen, Y. and Hsu, W.C., 2019. Effects of virtual reality-based physical and cognitive training on executive function and dual-task gait performance in older adults with mild cognitive impairment: a randomized control trial. Frontiers in aging neuroscience, 11, p.162.	Wrong outcome
Liao, Y.Y., Chen, I.H., Lin, Y.J., Chen, Y. and Hsu, W.C., 2019. Effects of virtual reality-based physical and cognitive training on executive function and dual-task gait performance in older adults with mild cognitive impairment: a randomized control trial. Frontiers in aging neuroscience, 11, p.162.	Wrong outcome
Mirelman, A., Rochester, L., Maidan, I., Del Din, S., Alcock, L., Nieuwhof, F., Rikkert, M.O., Bloem, B.R., Pelosin, E., Avanzino, L. and Abbruzzese, G., 2016. Addition of a non-immersive virtual reality component to treadmill training to reduce fall risk in older adults (V-TIME): a randomised controlled trial. The Lancet, 388(10050), pp.1170-1182.	Duplicate
Mirelman, A., Rochester, L., Maidan, I., Del Din, S., Alcock, L., Nieuwhof, F., Rikkert, M.O., Bloem, B.R., Pelosin, E., Avanzino, L. and Abbruzzese, G., 2016. Addition of a non-immersive virtual reality component to treadmill training to reduce fall risk in older adults (V-TIME): a randomised controlled trial. The Lancet, 388(10050), pp.1170-1182.	Duplicate
Mirelman, A., Rochester, L., Maidan, I., Del Din, S., Alcock, L., Nieuwhof, F., Rikkert, M.O., Bloem, B.R., Pelosin, E., Avanzino, L. and Abbruzzese, G., 2016. Addition of a non-immersive virtual reality component to treadmill training to reduce fall risk in older adults (V-TIME): a randomised controlled trial. The Lancet, 388(10050), pp.1170-1182.	Duplicate
Mirelman, A., Rochester, L., Maidan, I., Del Din, S., Alcock, L., Nieuwhof, F., Rikkert, M.O., Bloem, B.R., Pelosin, E., Avanzino, L. and Abbruzzese, G., 2016. Addition of a non-immersive virtual reality component to treadmill training to reduce fall risk in older adults (V-TIME): a randomised controlled trial. The Lancet, 388(10050), pp.1170-1182.	Duplicate
Mirelman, A., Rochester, L., Maidan, I., Del Din, S., Alcock, L., Nieuwhof, F., Rikkert, M.O., Bloem, B.R., Pelosin, E., Avanzino, L. and Abbruzzese, G., 2016. Addition of a non-immersive virtual reality component to treadmill training to reduce fall risk in older adults (V-TIME): a randomised controlled trial. The Lancet, 388(10050), pp.1170-1182.	Duplicate
Molhemi, F., Monjezi, S., Mehravar, M., Shaterzadeh-Yazdi, M.J., Salehi, R.,	Wrong

Hesam, S. and Mohammadianinejad, E., 2021. Effects of virtual reality vs conventional balance training on balance and falls in people with multiple sclerosis: a randomized controlled trial. Archives of physical medicine and rehabilitation, 102(2), pp.290-299.	population
Nuic, D., Van de Weijer, S., Cherif, S., Skrzatek, A., Zeeboer, E., Olivier, C., Corvol, J.C., Foulon, P., Pastor, J.Z., Mercier, G. and Lau, B., 2024. Home-based exergaming to treat gait and balance disorders in patients with Parkinson's disease: A phase II randomized controlled trial. European Journal of Neurology, 31(1), p.e16055.	Wrong outcome
Nuic, D., Van de Weijer, S., Cherif, S., Skrzatek, A., Zeeboer, E., Olivier, C., Corvol, J.C., Foulon, P., Pastor, J.Z., Mercier, G. and Lau, B., 2024. Home-based exergaming to treat gait and balance disorders in patients with Parkinson's disease: A phase II randomized controlled trial. European Journal of Neurology, 31(1), p.e16055.	Wrong outcome
Nuic, D., Van de Weijer, S., Cherif, S., Skrzatek, A., Zeeboer, E., Olivier, C., Corvol, J.C., Foulon, P., Pastor, J.Z., Mercier, G. and Lau, B., 2024. Home-based exergaming to treat gait and balance disorders in patients with Parkinson's disease: A phase II randomized controlled trial. European Journal of Neurology, 31(1), p.e16055.	Wrong outcome
Ayaz, P., Raza, Q., Tabba, M.A. and Baig, M.U., 2024. Evidence Synthesis on Gait and Balance Training: Meta-Analysis of Physical Therapy Intervention in Parkinson’s Patients: Physical Therapy in Parkinson Disease. Allied Medical Research Journal, 2(01), pp.256-268.	Wrong outcome
Bacanoiu, M.V., Rusu, L., Marin, M.I., Piele, D., Rusu, M.R., Danoiu, R. and Danoiu, M., 2025. Digital Health in Parkinson’s Disease and Atypical Parkinsonism—New Frontiers in Motor Function and Physical Activity Assessment. Journal of Clinical Medicine, 14(12), p.4140.	Wrong intervention
Song, J., Paul, S.S., Caetano, M.J.D., Smith, S., Dibble, L.E., Love, R., Schoene, D., Menant, J.C., Sherrington, C., Lord, S.R. and Canning, C.G., 2018. Home-based step training using videogame technology in people with Parkinson’s disease: a single-blinded randomised controlled trial. Clinical rehabilitation, 32(3), pp.299-311.	Duplicate
Song, J., Paul, S.S., Caetano, M.J.D., Smith, S., Dibble, L.E., Love, R., Schoene, D., Menant, J.C., Sherrington, C., Lord, S.R. and Canning, C.G., 2018. Home-based step training using videogame technology in people with Parkinson’s disease: a single-blinded randomised controlled trial. Clinical rehabilitation, 32(3), pp.299-311.	Duplicate
Stanmore, E.K., Mavroeidi, A., de Jong, L.D., Skelton, D.A., Sutton, C.J., Benedetto, V., Munford, L.A., Meekes, W., Bell, V. and Todd, C., 2019. The effectiveness and cost-effectiveness of strength and balance Exergames to reduce falls risk for people aged 55 years and older in UK assisted living facilities: a multi-centre, cluster randomised controlled trial. BMC medicine, 17(1), p.49.	Wrong outcome

Sturnieks, D.L., Hicks, C., Smith, N., Ratanapongleka, M., Menant, J., Turner, J., Lo, J., Chaplin, C., Garcia, J., Valenzuela, M.J. and Delbaere, K., 2024. Exergame and cognitive training for preventing falls in community-dwelling older people: a randomized controlled trial. Nature medicine, 30(1), pp.98-105.	Duplicate
Sturnieks, D.L., Hicks, C., Smith, N., Ratanapongleka, M., Menant, J., Turner, J., Lo, J., Chaplin, C., Garcia, J., Valenzuela, M.J. and Delbaere, K., 2024. Exergame and cognitive training for preventing falls in community-dwelling older people: a randomized controlled trial. Nature medicine, 30(1), pp.98-105.	Duplicate
Sturnieks, D.L., Menant, J. and Lord, S.R., 2022. Balance and ageing. In The biology of ageing (pp. 223-234). CRC Press.	Duplicate
Sturnieks, D.L., Hicks, C., Smith, N., Ratanapongleka, M., Menant, J., Turner, J., Lo, J., Chaplin, C., Garcia, J., Valenzuela, M.J. and Delbaere, K., 2024. Exergame and cognitive training for preventing falls in community-dwelling older people: a randomized controlled trial. Nature medicine, 30(1), pp.98-105.	Duplicate
Sturnieks, D.L., Hicks, C., Smith, N., Ratanapongleka, M., Menant, J., Turner, J., Lo, J., Chaplin, C., Garcia, J., Valenzuela, M.J. and Delbaere, K., 2024. Exergame and cognitive training for preventing falls in community-dwelling older people: a randomized controlled trial. Nature medicine, 30(1), pp.98-105.	Duplicate
van der Kolk, N.M., de Vries, N.M., Kessels, R.P., Joosten, H., Zwinderman, A.H., Post, B. and Bloem, B.R., 2019. Effects of a home-based aerobic exercise program in persons with Parkinson's disease, 18(11), p.115.	Duplicate
Van Schooten, K.S., Callisaya, M.L., O" Dea, B., Lung, T., Anstey, K., Lord, S.R., Christensen, H., Brown, A., Chow, J., McInerney, G. and Miles, L., 2021. Protocol of a 12-month multifactorial eHealth programme targeting balance, dual-tasking and mood to prevent falls in older people: the StandingTall+ randomised controlled trial. BMJ open, 11(4), p.e051085.	Wrong intervention
Wu, P.L., Lee, M., Wu, S.L., Ho, H.H., Chang, M.H., Lin, H.S. and Huang, T.T., 2021. Effects of home-based exercise on motor, non-motor symptoms and health-related quality of life in Parkinson's disease patients: A randomized controlled trial. Japan Journal of Nursing Science, 18(3), p.e12418.	Wrong intervention
Yalfani, A., Abedi, M. and Raeisi, Z., 2022. Effects of an 8-week virtual reality training program on pain, fall risk, and quality of life in elderly women with chronic low back pain: Double-blind randomized clinical trial. Games for Health Journal, 11(2), pp.85-92.	Wrong outcome
Zahedian-Nasab, N., Jaberi, A., Shirazi, F. and Kavousipor, S., 2021. Effect of virtual reality exercises on balance and fall in elderly people with fall risk: a randomized controlled trial. BMC geriatrics, 21(1), p.509.	Wrong outcome

Zhang, J., Luximon, Y., Pang, M.Y. and Wang, H., 2022. Effectiveness of exergaming-based interventions for mobility and balance performance in older adults with Parkinson's disease: systematic review and meta-analysis of randomised controlled trials. Age and ageing, 51(8), p.afac175.	Wrong outcome
Zougar, M., Todd, C., McGarrigle, L. and Stanmore, E., 2022. MIRA rehab exergames for older male residents in a care home center in Saudi Arabia: Protocol for a feasibility randomized controlled trial. JMIR Research Protocols, 11(12), p.e39148.	Wrong outcome

Supplementary File S3 - TIDieR table

Author, year	*Name of the intervention*	*WHY: Rationale or aims of the intervention*	*WHAT: Description of the intervention, including materials and activities*	*WHO: Stakeholders involved in developing the intervention*	*HOW: Modes of delivery*	*WHERE: Location of where the intervention is delivered/occurred, including infrastructure*	*HOW MUCH: Dose, Frequency, total duration of intervention*	*WHEN: Assessment/ follow-up points*	*TAILORING: Planned to be personalised or adapted (what why when how)*	*MODIFICATIONS: If modified, describe changes (what why when how)*	*HOW WELL: Adherence or fidelity, planned or actual*
Alagumoorthi 2022	Wii sports-based strategy training	Exercise interventions, including Wii fit training targeting postural control could improve the measures of risk of falling.	Materials: Nintendo Wii console<br><br>Activities: The training included six games from Wii Sports & two games from resort after analysing and validating the movement components. The games were selected to target different movements and strategies that required to avoid falls.	Experienced physiotherapists trained for specific protocol	Face-to-face	Participant's home	30-40 minutes per session, 3 times a week for 12 weeks	Before training, 12 weeks (post-training), 36 weeks (follow-up)	The games were selected to target different movements and strategies that required to avoid falls. Participants played the games in the order of their interest randomly. Safety harness belt were used to ensure safety of the participants	NA	The fall diary was used to identify and count the falls. Participants (or caregiver) kept the diary and recorded the number of falls they sustain on daily basis
Eggenberger 2015	Aerobic endurance with dance through VR (DANCE)	Simultaneous cognitive–physical training may create additional enhancements on DT gait variables	Materials: two Impact Dance Platforms (Positive Gaming BV, Haarlem, the Netherlands) and created various levels of difficulty in step patterns and frequency with the StepMania Software<br><br>Activities: Participants stood on the 1×1 m2 platform, which contained four pressure sensitive areas to detect steps forward, backward, to the left, and to the right, respectively. Stepping sequences were cued with arrows appearing on a large screen and had to be performed exactly when	Under instruction of two trained postgraduate students	Face-to-face; groups of five to six participants	Geriatrische Klinik, St Gallen, Switzerland	1 hour per session, twice a week for 6 months	Before training, 3 months, 6 months (post-training), 1-year (follow-up)	The cognitive–motor DT condition was adjusted to the participant's cognitive abilities. Participants were instructed not to prioritize either task and were allowed to use assistive walking devices.	NA	NR

			an arrow reached a highlighted area on the screen in order to achieve best scores in the game. Participants were instructed to hold on to ropes for security reasons.								
Fu 2015	Wii Fit balance training	Wii Fit platform offers feedback to the participants, enabling them to identify improved balance capabilities	Materials: Nintendo's Wii Fit balance board<br><br>Activities: Three balance training gamesdnamely, Soccer Heading, Table Tilt, and Balance Bubbledwere selected. These activities exercised various components of the balance control system, including musculoskeletal components, sensory systems, neuromuscular strategies, and anticipatory control.	A physiotherapist conducted the whole training regime for all participants. Because all the participants had history of falls, they were accompanied by a rehabilitation assistant who provided immediate manual support when necessary during both the Wii Fit and the conventional balance training programs.	Face-to-face	Nursing homes	1 hour per session, three times a week for 6 weeks	Before training, 12 months (follow-up)	Players progress to the harder mode of the game at their own pace. This pace was determined through the game's "star system" that rates the player's performance on each individual game. Participants were rested while each game was being restarted. Because all the participants had history of falls, they were accompanied by a rehabilitation assistant who provided immediate manual support when necessary during both the Wii Fit and the conventional balance training programmes.	NA	Fall incidence was recorded by the nursing staff according to the aforementioned definition and reported to the investigator for each participant monthly over the 12-month period after randomization. Nurses at the nursing home who documented falls were unaware of participants' group allocation.
Gandolfi 2017	TeleWii-Lab	Remotely supervised home-based virtual reality telerehabilitation to compare improvements in postural stability	Materials: A TeleWii-Lab comprising the Nintendo Wii console for motion controlled inputs, the Wii Fit gaming system, and balance board was set up at each rehabilitation unit. A laptop computer connected to a high-resolution web-camera was used to establish realtime remote visual communication via Skype software (Skype/Microsoft) between the rehabilitation unit and the	A physiotherapist gave a full explanation of the training protocol and conducted a trial TeleWii session at the hospital lab. During each session, the physiotherapist supervised two patients simultaneously. A caregiver	Remote	Four neurorehabilitation units, Patient's home	50 minutes per session, 3 times per week for 7 consecutive weeks (21 sessions)	Before training, after training, and 1-month (follow-up)	TeleWii training included the following 10 exergames selected by the physiotherapist according to the patient's clinical condition and progressive improvement over time.	NA	The number of falls in the previous month was recorded in a self-report log. Patients were provided with logbook to record their feelings and any difficulties or adverse events they had experienced at each training session.

			patient's home. A research team member installed an identical TeleWii set-up at the patient's home.<br><br>Activities: In-home TeleWii training consisted of 21 sessions of balance exercises of 50 minutes each. A brief warm-up consisted of stretching exercises of the upper and lower extremities. TeleWii training included 10 exergames selected by the physiotherapist according to the patient's clinical condition and progressive improvement over time. The Skype video calls lasted the entire duration of each training session.	was always present to monitor the patient during training and warrants its safety.							
Kwok 2016	Nintendo Wii exercise programme	Nintendo Wii exercise programme can improve confidence, strength, physical function and reduce sedentary behaviour in older adults	Materials: Nintendo Wii Active<br><br>Activities: Nintendo WiiActive gaming exercises with the Wii balance board and resistance band, which included cardiovascular training, resistance band strengthening, calisthenics and balance training. 20 min Wii intervention + 20 min participant-specific interventions + 20 min home exercise	Each intervention session was supervised by a physiotherapist and a therapist assistant.	Face-to-face, group of four to six participant s	Singapore General Hospital (SGH) outpatient centres	1 hour per session, 1 hour (2-3 days) for 12 weeks	Before training, week 13 (post-training), week 24 (follow-up)	To ensure participants' safety, a firm chair was placed in front of the participants when they performed balance activities on the Wii balance board under supervision.	NA	Treatment compliance was monitored by (i) the number of intervention sessions attended and (ii) the frequency of home exercises performed (recorded in a log sheet).
Mirelman 2016	Treadmill-based intervention with VR (V-TIME)	The virtual environment was specifically designed to reduce fall risk in older	Materials: the system included a camera for motion capture (a modified Microsoft Kinect for Windows, Microsoft, Redmond, WA, USA) and a computer	A trainer was present at all training sessions. Research staff contacted all participants	Face-to-face	Five clinical centere across five countries	45 minutes per session, 3 times a week for 6 weeks	(1 week) Before training, 1 week after intervention (post-training), 6	Training progression was structured in accordance with a prespecified plan for progression and was based on increasing both motor and cognitive challenges that were	Deviations from the original protocol16 were widening of the age range from 60−85 years to	Research staff contacted all participants every month to maximise compliance. Fall rate was recorded during the 6 months after the end of training. Participants

		adults	generated simulation<br><br>Activities: The camera recorded the movement of the participant's feet while they walked on the treadmill. These images were projected to the participant in real time on a large screen during the training, enabling the participants to see their feet walking within the simulation. The virtual environment imposed a cognitive load that demands attention, planning, dual tasking, response selection, and processing of rich auditory and visual stimuli that involve several perceptual processes.	every month to maximise compliance.				months (follow-up)	individualised to the participant's level of performance. Progression of the intervention was modulated via the speed of the treadmill, the duration of the walking bouts within a given training session, and the size and frequency of the virtual obstacles and the distractors.	60−90 years to allow for inclusion of additional participants who could benefi t from the interventions; lowering of the MMSE cutoff score from more than 24 to more than 21 to include participants with a wider range of cognitive impairments;and removal of the exclusion cutoff based on the New Freezing of Gait questionnaire, because they realised that the existence of freezing of gait did not negate training.	received a falls calendar, which they were provided as a paper version, web-based calender, or a smartphone application in accordance with their preference. Information logged in the online or smartphone-based calendar was automatically uploaded to a database, whereas the paper calendars were posted back to the sites at which participants were recruited each month via pre-addressed envelopes. The falls database was checked, reviewed, and locked before intervention group assignment was unmasked
Song 2018	Home-based Exergame step training	This form of step training has the potential to provide engaging and relatively inexpensive exercise that people with Parkinson's disease could undertake at home at times convenient to them	Materials: Dance Dance Revolution Stepmania<br><br>Activities: Participants were instructed to stand on the central stance panels and step on the appropriate target arrow (right, left, right front or back and left front or back) to match the direction and timing (varying stimulus speed) of the arrows that were drifting from the bottom to the top of the television screen. After	Participants in the intervention group were taught to perform the exergame in their home by an experienced physiotherapist .	Face-to-face	Home visit	15 minutes per session, three times a week for 12 weeks	Before training, 12 weeks (post-training)	The step training game had four levels of difficulty: novice, easy, medium and hard. All participants started with the easy level at the first home visit. At the second home visit, the physiotherapist modified the level as appropriate based on participants' performance and then participants were instructed to progress or regress the level of difficulty as appropriate.	The exergame was a modified version of the open-source Dance Dance Revolution "Stepmania" game (www.stepmania.com)	Falls were recorded prospectively for six months via monthly falls diaries. Participants received a monthly phone call to confirm any reported falls and to provide reminders to mail the diaries when required.24 Adverse events and medications were also monitored and recorded throughout the study via these standardised calls.

			each step, participants were given feedback in the form of a word in the centre of the screen (perfect, good, miss) and accrued points were presented on the screen after finishing each game.								
Stanmore 2019	Exergame (a tailored strength and balance Exergame programme)	This Exergame system showed potential to improve balance and increased engagement through motivational design.	Materials: Microsoft Kinect, a 3D motion tracking device that does not require handheld controls. This tracks the user's performance and records parameters such as frequency and duration of use.<br><br>Activities: Each participant was given a prescribed programme of standardised Exergames that suited the participant's starting level of ability, with tailored progression (e.g. more exergames within a session, greater challenge, longer duration).	Exergames were under the supervision of a physiotherapist or physiotherapist assistant.	Face-to-face	18 assisted living (sheltered housing) facilities (clusters)	30 minutes per session, 3 times a week for 12 weeks	Before training, 12 weeks (post-training)	Individual exercise programmes can be tailored using a choice of games for lower or upper limb exercises.	NA	A 3-month follow-up of self-reported participant falls (baseline assessment) was conducted using daily fall calendars that were posted monthly to the researchers. Participants who reported a fall during the previous month were contacted by telephone to record details of the fall.
Sturnieks 2024	Smart±step programme	Interactive computer games can provide cognitive training while providing motivating characteristics such as entertaining activities, game challenges, immediate feedback and high score targets to maximise	Materials: Desktop touchpad or step mat. Smart±step computer game system; a personal computer running custom software providing eight games, displayed on a television or computer screen.<br><br>Activities: The games required players to move in different directions and challenged speed, accuracy and motor control, and targeted specific cognitive functions including	The intervention groups received a home visit from an Exercise Physiology-trained staff member (Trainer) who provided and installed the necessary equipment in a safe and practical location in the participant's homes and	Face-to-face	Participant's home	120-150 minutes per week for 12 months	Before training, 12 months (post-training)	Exergame difficulty levels were subsequently chosen by participants for the remainder of the trial	NA	Adherence to the interventions was monitored via automatic daily data transfer from each participant's smart±step personal computer to a central server. Participants who were engaging in less than 80 minutes of training per week for two consecutive weeks (who had not informed the team of absence or illness) were contacted by telephone by the Trainer to encourage improved participation, assist with

		adherence.	working memory, visuospatial skills, dual-tasking, inhibition and attention. The cognitive-only training group played the games using their hands while seated and using a custom-built desktop touch pad with touch sensing targets corresponding to forward, backward, left and right moves. The cognitive-motor training group played the same games while standing and stepping onto a Bluetooth connected (wireless) step mat also with sensing targets corresponding to forward, backward, left and right moves, around a centre standing position.	provided exercise and system use instructions.							goal setting and help address any barriers to training. Participants reported falls using monthly calendars for daily recording and monthly return to research staff via email or postal mail.

Supplementary File S4 - GRADE assessments

Summary of findings:

Exergaming compared to usual care or no intervention for older people and falls

Patient or population: older people and falls

Setting: Community

Intervention: Exergaming

Comparison: usual care or no intervention

Outcomes	Anticipated absolute effects* (95% CI)		Relative effect (95% CI)	№ of participants (studies)	Certainty of the evidence (GRADE)	Comments
	**Risk with usual care or no intervention**	**Risk with Exergaming**				
Number of falls (Falls)<br>follow-up: range 12 weeks to 12 months		**0 per 1,000**<br>(0 to 0)	**IRR 0.55**<br>(0.47 to 0.65)	652<br>(3 RCTs)	⨁⨁◯◯<br>Low[a,b,c,d]	Exergaming may result in a reduction in the fall rate
Number of Fallers (Number of fallers)<br>assessed with: Count<br>follow-up: range 12 weeks to 12 months	not pooled	not pooled	not pooled	652<br>(3 RCTs)	⨁⨁◯◯<br>Low[e,f,g]	Exergaming probably results in a slight reduction in number of Fallers.
No. of people who experienced one or more fall-related injuries (One or more injuries)<br>assessed with: count<br>follow-up: mean 12 months	310 per 1,000	**251 per 1,000**<br>(331 to 189)	**RR 0.81**<br>(0.61 to 1.07)	507<br>(1 RCT)	⨁⨁◯◯<br>Low[h]	Exergaming may slightly reduce the number of people who experienced one or more fall-related injuries.
HR-QoL (HR-QoL)		MD **3.4 higher**<br>(5.6 lower to 12.4 higher)	-	92<br>(1 RCT)	⨁⨁◯◯<br>Low[i]	Exergaming may increase HR-QoL.
Concerns about Falling (Concerns about Falling)<br>follow-up: range 12 weeks to 12 months	not pooled	not pooled	-	652<br>(3 RCTs)	⨁⨁◯◯<br>Low[j,k]	Exergaming may result in little to no difference in concerns about Falling.

***The risk in the intervention group** (and its 95% confidence interval) is based on the assumed risk in the comparison group and the **relative effect** of the intervention (and its 95% CI).

CI: confidence interval; **MD:** mean difference; **RR:** risk ratio; **SMD:** standardised mean difference

Summary of findings:

Exergaming compared to usual care or no intervention for older people and falls

Patient or population: older people and falls

Setting: Community

Intervention: Exergaming

Comparison: usual care or no intervention

Outcomes	Anticipated absolute effects* (95% CI)		Relative effect (95% CI)	№ of participants (studies)	Certainty of the evidence (GRADE)	Comments
	**Risk with usual care or no intervention**	**Risk with Exergaming**				

GRADE Working Group grades of evidence
High certainty: we are very confident that the true effect lies close to that of the estimate of the effect.
Moderate certainty: we are moderately confident in the effect estimate: the true effect is likely to be close to the estimate of the effect, but there is a possibility that it is substantially different.
Low certainty: our confidence in the effect estimate is limited: the true effect may be substantially different from the estimate of the effect.
Very low certainty: we have very little confidence in the effect estimate: the true effect is likely to be substantially different from the estimate of effect.

Explanations

a. Two studies have some risk of bias concerns relating to blinding of participants and assessors. It was not possible to blind participants to the intervention, so this was not considered serious

b. Effect in same direction but CI for Song crosses line of no effect

c. The CI for Stunieks study are almost outside CI for Stanmore study

d. CI for Song crosses line of no effect and Song and Stanmore have small sample sizes

e. All studies re at risk of bias from non blinding of participants and researchers, but this is because it is not possible to conceal intervention. This is not considered to be important

f. All outcomes are in same direction, but CI for Stanmore and Song cross line of no effect.

g. Outcome at 3 and 6 months was not significant. Significant effect at 12 months

h. Only one study

i. CI crosses line of no effect, wide confidence intervals and small sample size

j. High heterogeneity I2 = 73%. 3/4 outcome measurements are in same direction, but one is in opposite direction.

k. Two studies cross line of no effect. Wide CI for some studies

Exergaming compared to Active control (evidence-based) for older people and falls

Patient or population: Older people and falls **Setting:** Community **Intervention:** Exergaming **Comparison:** Active control (evidence based)

Outcomes	**Anticipated absolute effects*** (95% CI)		Relative effect (95% CI)	№ of participants (studies)	Certainty of the evidence (GRADE)	Comments
	**Risk with Active control (evidence based)**	**Risk with Exergaming**				
Rate of Falls follow-up: range 1 months to 12 months		**0 per 1,000** (0 to 0)	**IRR 0.37** (0.30 to 0.45)	130 (2 RCTs)	⊕⊕◯◯ Low[a,b]	Exergaming probably results in a slight reduction in the fall rate
Number of Fallers	200 per 1,000	**276 per 1,000** (124 to 612)	**RR 1.38** (0.62 to 3.06)	80 (1 RCT)	⊕⊕◯◯ Low[a,c]	Exergaming probably results in a slight reduction in number of fallers.
HR-QoL		MD **1.91 higher** (4.67 lower to 8.49 higher)	-	70 (1 RCT)	⊕⊕◯◯ Low[a]	Exergaming probably results in an increase in hR-QoL.
Concerns about Falling		MD **0.8 lower** (1.44 lower to 0.26 lower)	-	53 (1 RCT)	⊕⊕◯◯ Low[a]	Exergaming may reduce concerns about Falling.
Balance confidence		MD **4.61 higher** (3.88 lower to 13.1 higher)	-	70 (1 RCT)	⊕⊕◯◯ Low[a]	Exergaming likely results in an increase in balance confidence.
Acceptance follow-up: range 1 months to 12 months	-	SMD **0.19 SD higher** (0.16 lower to 0.53 higher)	-	134 (2 RCTs)	⊕⊕◯◯ Low[d]	The evidence suggests that exergaming does not reduce acceptance.
Adherence (home exercise) follow-up: 52 weeks		MD **0.3 lower** (0.94 lower to 0.34 higher)	-	64 (1 RCT)	⊕⊕◯◯ Low[c]	Exergaming may result in little to no difference in adherence (home exercise).

***The risk in the intervention group** (and its 95% confidence interval) is based on the assumed risk in the comparison group and the **relative effect** of the intervention (and its 95% CI).

CI: confidence interval; **MD:** mean difference; **RR:** risk ratio; **SMD:** standardised mean difference

GRADE Working Group grades of evidence
High certainty: we are very confident that the true effect lies close to that of the estimate of the effect.
Moderate certainty: we are moderately confident in the effect estimate: the true effect is likely to be close to the estimate of the effect, but there is a possibility that it is substantially different.
Low certainty: our confidence in the effect estimate is limited: the true effect may be substantially different from the estimate of the effect.
Very low certainty: we have very little confidence in the effect estimate: the true effect is likely to be substantially different from the estimate of effect.

Explanations

a. CI crosses line of no effect
b. Both studies were small with sample sizes of approximately 30-35 participants
c. small sample size with wide CI
d. small study sizes

Exergaming compared to Active control (non-evidence based) for older people and falls

Patient or population: older people and falls **Setting: Community** **Intervention:** Exergaming **Comparison:** Active control (non-evidence based)

Outcomes	Anticipated absolute effects* (95% CI)		Relative effect (95% CI)	№ of participants (studies)	Certainty of the evidence (GRADE)	Comments
	**Risk with Active control (non-evidence based)**	**Risk with Exergaming**				
Rate of Falls<br>follow-up: range 24 weeks to 52 weeks		**0 per 1,000**<br>(0 to 0)	**IRR 0.57**<br>(0.48 to 0.67)	665<br>(3 RCTs)	⊕⊕⊕◯<br>Moderate[a,b]	Exergaming probably results in a slight reduction in rate of falls
Number of fallers<br>follow-up: range 24 weeks to 52 weeks	Not pooled	Not pooled		516<br>(3 RCTs)	⊕⊕◯◯<br>Low[c,d]	Exergaming may reduce the number of fallers slightly.
Health related quality of life (HR-QoL)<br>follow-up: range 24 weeks to 52 weeks	-	Not pooled	-	471<br>(2 RCTs)	⊕⊕⊕◯<br>Moderate[b]	Exergaming may result in little to no difference in hR-QoL .
Concerns about Falling<br>follow-up: range 24 weeks to 52 weeks		MD **1.6 higher**<br>(0.63 lower to 3.83 higher)	-	30<br>(1 RCT)	⊕◯◯◯<br>Very low[d]	The evidence is very uncertain about the effect of exergaming on concerns about falling
Adherence<br>follow-up: 6 months		MD **0.2 higher**<br>(0.22 lower to 0.62 higher)	-	282<br>(1 RCT)	⊕⊕◯◯<br>Low[e]	Exergaming may result in little to no difference in adherence.

***The risk in the intervention group** (and its 95% confidence interval) is based on the assumed risk in the comparison group and the **relative effect** of the intervention (and its 95% CI).

CI: confidence interval; **MD:** mean difference; **RR:** risk ratio; **SMD:** standardised mean difference

GRADE Working Group grades of evidence
High certainty: we are very confident that the true effect lies close to that of the estimate of the effect.
Moderate certainty: we are moderately confident in the effect estimate: the true effect is likely to be close to the estimate of the effect, but there is a possibility that it is substantially different.
Low certainty: our confidence in the effect estimate is limited: the true effect may be substantially different from the estimate of the effect.
Very low certainty: we have very little confidence in the effect estimate: the true effect is likely to be substantially different from the estimate of effect.

Explanations

a. Low heterogeneity
b. Point estimates pass through null value and sample sizes are small
c. I2 moderate
d. Point estimates pass through null value and sample sizes small
e. Relatively small sample size
